# Lipoprotein(a) and residual cardiovascular risks in Japanese patients with coronary artery disease who achieve guideline-recommended low-density lipoprotein cholesterol goals

**DOI:** 10.1093/eurheartj/ehag446

**Published:** 2026-05-24

**Authors:** Yu Kataoka, Kausik K Ray, Stephen J Nicholls, Aya Katasako-Yabumoto, Satoshi Kitahara, Hayato Hosoda, Kentaro Mitsui, Kota Murai, Kenichiro Sawada, Hideo Matama, Takamasa Iwai, Satoshi Honda, Masashi Fujino, Shuichi Yoneda, Kazuhiro Nakao, Kensuke Takagi, Yasuhide Asaumi, Teruo Noguchi

**Affiliations:** Department of Cardiovascular Medicine, National Cerebral & Cardiovascular Center, Suita, Japan; Department of Primary Care and Public Health, School of Public Health, Imperial College London, London, UK; Medicine Monash Health, Victorian Heart Institute (VHI), Clayton, Australia; Department of Cardiovascular Medicine, National Cerebral & Cardiovascular Center, Suita, Japan; Department of Cardiovascular Medicine, Kashiwa Kousei General Hospital, Kashiwa, Japan; Department of Cardiovascular Medicine, Chikamori Hospital, Kochi, Japan; Department of Cardiovascular Medicine, National Cerebral & Cardiovascular Center, Suita, Japan; Department of Cardiovascular Medicine, National Cerebral & Cardiovascular Center, Suita, Japan; Department of Cardiovascular Medicine, National Cerebral & Cardiovascular Center, Suita, Japan; Department of Cardiovascular Medicine, National Cerebral & Cardiovascular Center, Suita, Japan; Department of Cardiovascular Medicine, National Cerebral & Cardiovascular Center, Suita, Japan; Department of Cardiovascular Medicine, National Cerebral & Cardiovascular Center, Suita, Japan; Department of Cardiovascular Medicine, National Cerebral & Cardiovascular Center, Suita, Japan; Department of Cardiovascular Medicine, National Cerebral & Cardiovascular Center, Suita, Japan; Department of Cardiovascular Medicine, National Cerebral & Cardiovascular Center, Suita, Japan; Department of Cardiovascular Medicine, National Cerebral & Cardiovascular Center, Suita, Japan; Department of Cardiovascular Medicine, National Cerebral & Cardiovascular Center, Suita, Japan; Department of Cardiovascular Medicine, National Cerebral & Cardiovascular Center, Suita, Japan

**Keywords:** Lipoprotein (a), Low-density lipoprotein cholesterol, Coronary artery disease, Atherosclerotic cardiovascular disease

## Abstract

**Background and Aims:**

Current guidance recommends better LDL-C control in patients with elevated Lp(a) as Lp(a) lowering therapies are unavailable. Whether risks attributable to Lp(a) are mitigated in patients with CAD who achieved LDL-C <55 mg/dL remains unknown.

**Methods:**

Multicentre retrospective observational study analysed 1581 Japanese patients with CAD [Lp(a)-JAPAN: jRCT1050260016]. Risk of MACE (cardiac-caused death + nonfatal MI + coronary revascularization in non-culprit segments) was compared according to Lp(a) levels (<30, ≥30, and <50 mg/dL and ≥50 mg/dL) and amongst LDL-C strata (<55 mg/dL vs ≥55 mg/dL) 8 weeks after PCI.

**Results:**

During the 5.1-year observation amongst patients with LDL-C ≥55mg/dL (n=1069), MACE occurred in 21.3% with risk of MACE increasing with Lp(a) levels (3.9, 7.9, and 11.0 events per 100 person-years for <30, ≥30, and <50 mg/dL and ≥50 mg/dL, log-rank p<0.001). Amongst those with LDL-C <55mg/dL (n=512), the proportion with MACE was lower overall (4.3%, p<0.001). However, elevated Lp(a) levels still identified those at higher risk of MACE (1.4, 4.7, and 7.5 events per 100 person-years in Lp(a) <30, ≥30, and <50 mg/dL and ≥50 mg/dL, p<0.001). Standardized 5-year MACE rate was over twice and five times higher in patients with Lp(a) ≥30 and <50 mg/dL (17.0%: adjusted HR = 3.80, 95% CI = 1.78–8.11, p<0.001) and ≥50 mg/dL (33.4%: adjusted HR = 6.90, 95% CI = 3.53–13.46, p<0.001) compared to Lp(a) <30 mg/dL (5.0%), respectively. The ROC analyses identified Lp(a) ≥28.2 mg/dL as the threshold for MACE (AUC = 0.68, p<0.001).

**Conclusions:**

Whilst lower achieved LDL-C attenuates in part the risk from Lp(a), elevated Lp(a) levels still associate with worse cardiovascular outcomes. The Lp(a) threshold amongst Japanese patients with CAD at risk of recurrent events appears lower than in Caucasian populations which merits further evaluation.

## Structured Graphical Abstract

**Structured Graphical Abstract:**
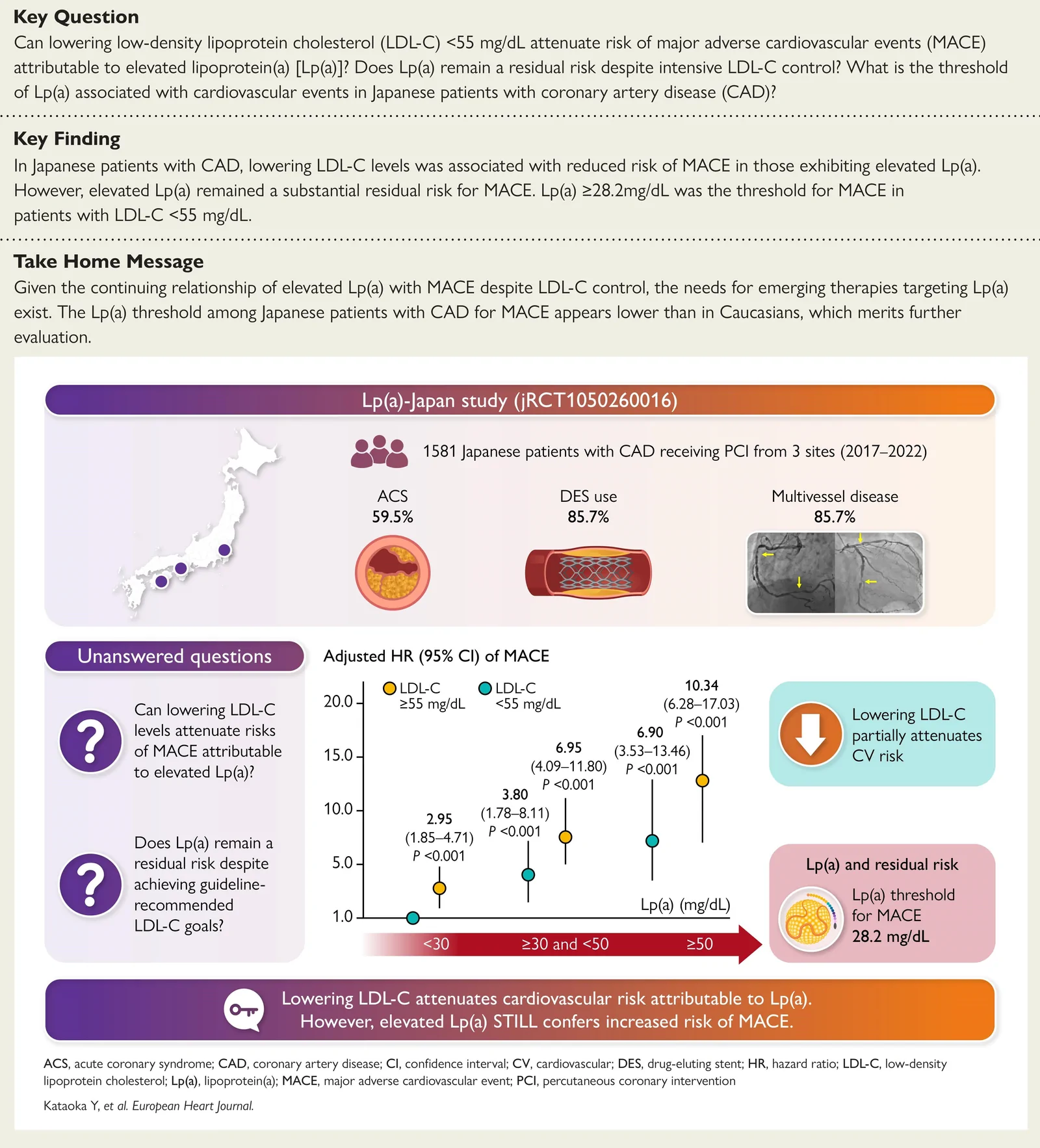

**See the editorial comment for this article ‘Lipoprotein(a) in Japan: when ‘moderately elevated' might already mean high risk’, by F. Kronenberg, https://doi.org/10.1093/eurheartj/ehag607.**

## Introduction

Circulating lipoprotein(a) [Lp(a)] is an atherogenic cholesterol-containing lipoprotein which promotes the formation and progression of atherosclerosis, ultimately causing atherosclerotic cardiovascular disease (ASCVD).^1,2^ Epidemiological and genetic studies have consistently reported Lp(a) as an independent, causal risk factor for ASCVD in several ethnic groups.^3–7^ This evidence supports the proatherogenic properties of Lp(a) and the potential need for additional therapies to mitigate risk of ASCVD attributable to Lp(a). However, the risk attributable to Lp(a) among patients with coronary artery disease (CAD) may vary by ethnicity.^8,9^ Moreover, median Lp(a) levels among patients with CAD vary widely, suggesting that different populations may be more or less vulnerable to the same Lp(a) level.^8,9^ Since specific Lp(a)-lowering therapies are not established yet, the current European Atherosclerosis Society (EAS) consensus statement and European Society of Cardiology (ESC) 2025 Update to the 2019 dyslipidaemia guidelines recommend lowering low-density lipoprotein cholesterol (LDL-C) levels and other risk factors.^10,11^

Low-density lipoprotein cholesterol is a highly modifiable risk factor, and lowering LDL-C slows the progression of atherosclerosis and reduces ASCVD risk. Recent randomized controlled trials and observational studies have demonstrated reduced cardiovascular risk among those achieving very low LDL-C levels in very high-risk patients including those with CAD.^12–17^ Hence, the ESC/EAS guideline advocates an LDL-C <55 mg/dL for the secondary prevention of ASCVD.^11,18^ However, whether achieving this stringent goal of LDL-C levels sufficiently mitigates increased risks attributable to an elevated Lp(a) level remains to be fully elucidated among Japanese patients with most data arising from Caucasian populations. Therefore, the current study investigated whether elevated Lp(a) levels contribute to cardiovascular outcomes in Japanese patients with CAD and to what extent lowering LDL-C <55 mg/dL mitigates this risk.

## Methods

### Study participants

This study was a multicentre, observational study conducted at three sites in Japan [Lp(a) and Cardiovascular Outcomes following Guideline Recommended LDL-C Control in JAPANese Patients with CAD: Lp(a)JAPAN study—jRCT1050260016]. The following patients were included: (i) those with CAD, (ii) those with a clinical indication for percutaneous coronary intervention (PCI), (iii) those with measurement of LDL-C and Lp(a) levels at 2 months after PCI, and (iv) those with clinical follow-up for at least 3 years after PCI. The exclusion criteria were (i) CAD attributable to non-atherosclerotic causes, (ii) no measurement of LDL-C or Lp(a) levels at 2 months after PCI, (iii) no clinical follow-up data at 3 years after PCI, and (iv) major adverse cardiovascular events (MACE) within 2 months after PCI. From 1 January 2017 to 31 August 2022, a total of 3516 patients with CAD from three sites (National Cerebral & Cardiovascular Center, Kashiwa Kousei General Hospital, and Chikamori Hospital) were identified. Of these, 1581 patients were included in the current analysis (*Figure 1*). The study protocol conformed with the ethical guidelines of the 1975 Declaration of Helsinki. This study was approved by the institutional review board of the National Cerebral and Cardiovascular Center (R21053).

**Figure 1:**
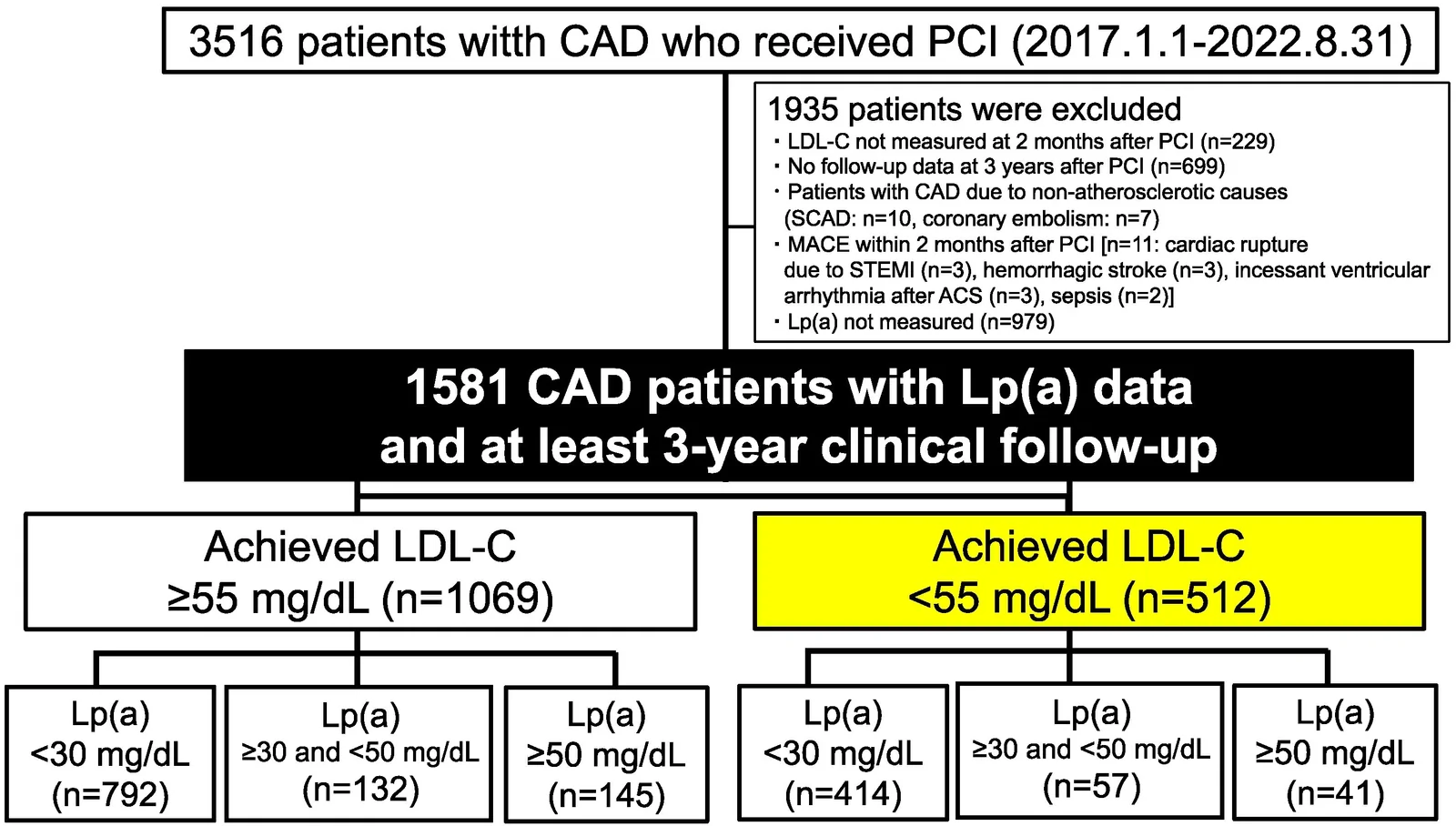
Patients’ disposition. To convert lipid values to SI units: LDL-C, multiply by .02586 (mg/dL to mmol/L); Lp(a), multiply by 2.381 (mg/dL to mmol/L). CAD, coronary artery disease; LDL-C, low-density lipoprotein cholesterol; Lp(a), lipoprotein(a); MACE, major adverse cardiovascular event; PCI, percutaneous coronary intervention

### Lipid-lowering therapies and measurements of lipid parameters

In the current study, following the completion of PCI, both LDL-C and Lp(a) levels were evaluated at 2 months after PCI. This LDL-C level was defined as ‘achieved LDL-C level’, which was used to stratify the study participants. Low-density lipoprotein cholesterol levels were calculated using the Friedewald formula. In patients with triglyceride levels >400 mg/dL, direct method (Sekisui Medical, Tokyo, Japan) was used to measure LDL-C levels. Control of LDL-C was conducted according to the guidelines from the 2022 Japanese Circulation Society and the 2022 Japan Atherosclerosis Society.^19,20^ The addition of ezetimibe and/or proprotein convertase subtilisin/kexin Type 9 inhibitor (PCSK9-I) was considered when LDL-C goal was not achievable with a statin [LDL-C goal: <100 mg/dL in patients with CAD, <70 mg/dL in patients with acute coronary syndrome (ACS), Type 2 diabetic patients with CAD, those with CAD and familial hypercholesterolaemia].^19^ High-intensity statin was defined as either atorvastatin ≥20 mg, rosuvastatin ≥10 mg, or pitavastatin ≥4 mg.^20^ All lipid parameters were measured in the fasting state. Lipoprotein(a) and serum levels of triglycerides and high-density lipoprotein cholesterol (HDL-C) were measured by enzymatic methods (Sekisui Medical, Tokyo, Japan) using an automated analyser (Hitachi Labospect 008; Hitachi-Hitec, Tokyo, Japan). All lipid values are expressed in mg/dL. To convert to SI units, the following conversion factors should be applied—LDL-C and HDL-C: multiply by .02586 to convert mg/dL to mmol/L; triglycerides: multiply by .01129 to convert mg/dL to mmol/L; and Lp(a): multiply by 2.381 to convert mg/dL to nmol/L using an average molecular weight of 420 000 Da, although this conversion is approximate given the size heterogeneity of apolipoprotein(a) isoforms.^21,22^

### Clinical follow-up after PCI

The study patients were followed up in outpatient clinics at each institute, or by a general practitioner, or both every 3–6 months according to the 2019 ESC guideline.^23^ Noninvasive testing for myocardial ischaemia after PCI was conducted according to suggestive angina chest pain, electrocardiogram/echocardiography abnormalities, elevation of cardiac enzymes, and/or the discretion of each physician.^24^ Invasive coronary angiography was performed based on findings from the aforementioned test. In patients with angiographical intermediate stenosis, myocardial ischaemia was evaluated by measurement of fractional flow reserve with pressure-monitoring guidewire.^25^ Fractional flow reserve ≤.80 was considered as an indication for revascularization.^26–28^

### Outcomes

The primary outcome was the occurrence of MACE, which included a composite of cardiac death, nonfatal myocardial infarction (MI), and clinically driven coronary revascularization at non-culprit segments. The secondary outcome was each component of MACE. Clinically driven coronary revascularization at non-culprit segments was defined as PCI or coronary artery bypass graft surgery due to the progression of non-culprit segments causing myocardial ischaemia/necrosis. This endpoint did not include coronary revascularization due to in-stent restenosis and/or stent thrombosis. These outcomes were obtained by reviewing medical records. If needed, the questionnaire was administered *via* mail or telephone. A clinical event committee consisting of physicians (KM and AK) and another referee (MF) adjudicated all events based on the aforementioned original source documents of outcomes.

### Statistical analysis

A detailed description of the statistical methods is provided in the Supplementary Material.

Continuous variables with a reasonably symmetrical distribution are presented as mean (standard deviation), and variables with skewed distributions are presented as median (interquartile range). Categorical variables are presented as numbers and percentages. Given the ordinal nature of Lp(a) categories, all between-group comparisons were performed using the Jonckheere–Terpstra test for continuous variables and the Cochran–Armitage test for categorical variables.

Survival was analysed using the Kaplan–Meier method. The association between Lp(a) levels and MACE was assessed using multivariable Cox proportional hazards models. The primary analysis used a six-category exposure variable defined by the combination of Lp(a) levels (<30, ≥30 and <50, and ≥50 mg/dL) and achieved LDL-C strata (<55 and ≥55 mg/dL), with patients having Lp(a) <30 mg/dL and achieved LDL-C <55 mg/dL as the reference group. The model was adjusted for age, sex, hypertension, Type 2 diabetes mellitus, prior MI, and multivessel disease, with stratification by ACS, dyslipidaemia, and chronic kidney disease (CKD) to address nonproportional hazards. Confounders were selected based on the disjunctive cause criterion, as illustrated in a directed acyclic graph (DAG) (Supplementary Figure I). Standardized 5-year MACE rates were estimated using Cox regression-based marginal standardization, and pairwise comparisons between groups were performed using bootstrap-based testing (500 resamples), with *P*-values adjusted for multiple comparisons using the Holm method. Adjusted hazard ratios (HRs) with 95% confidence interval (CI) were estimated from the same model, and pairwise comparisons were performed using separate Cox models for each pair, with *P*-values similarly adjusted using the Holm method. Multiplicative and additive interactions between Lp(a) and achieved LDL-C were formally assessed using the likelihood ratio test and the relative excess risk due to interaction (RERI), respectively. Sensitivity analyses included a stratified Cox model with stratification by achieved LDL-C levels and restricted cubic spline analysis. As this was a retrospective observational study using an existing registry, a formal *a priori* sample size calculation was not performed. The adequacy of the sample size is reflected by the precision of the multivariable-adjusted effect estimates: in patients with achieved LDL-C ≥55 mg/dL, the adjusted HRs for MACE were 2.30 (95% CI 1.61–3.28) for Lp(a) ≥30 and <50 mg/dL vs. <30 mg/dL and 3.51 (95% CI 2.59–4.76) for Lp(a) ≥50 vs. <30 mg/dL, indicating adequate precision. In patients with achieved LDL-C <55 mg/dL, the adjusted HRs for MACE were 3.64 (95% CI 1.68–7.92) for Lp(a) ≥30 and <50 mg/dL vs. <30 mg/dL and 5.96 (95% CI 3.05–11.67) for Lp(a) ≥50 vs. <30 mg/dL; while the CIs were wider reflecting the smaller number of events in this subgroup, the estimates remained statistically significant and directionally consistent with the primary analysis. All statistical analyses were performed using R version 4.5.1 (R Foundation for Statistical Computing, Vienna, Austria).

## Results

Overall among 1581 patients included, the average age was 68.3 ± 12.4 years old and 23.6% were female. *Figure 2A* illustrates the distribution of Lp(a) and LDL-C levels among the overall cohort. The median level of Lp(a) was 12.8 mg/dL (interquartile range: 6.3–29.4 mg/dL) and mean LDL-C was 59 ± 12 mg/dL. The proportion of patients with Lp(a) < 30 mg/dL, ≥30 and <50 mg/dL, and *≥*50 mg/dL overall were 76.3% (1206 of 1581), 11.9% (189 of 1581), and 11.8% (186 of 1581), respectively. *Figures 2B and 2C* show the distribution of Lp(a) among those with LDL-C above or below 55 mg/dL at 1.9 months (interquartile range: 1.5–2.3 months) after PCI.

**Figure 2:**
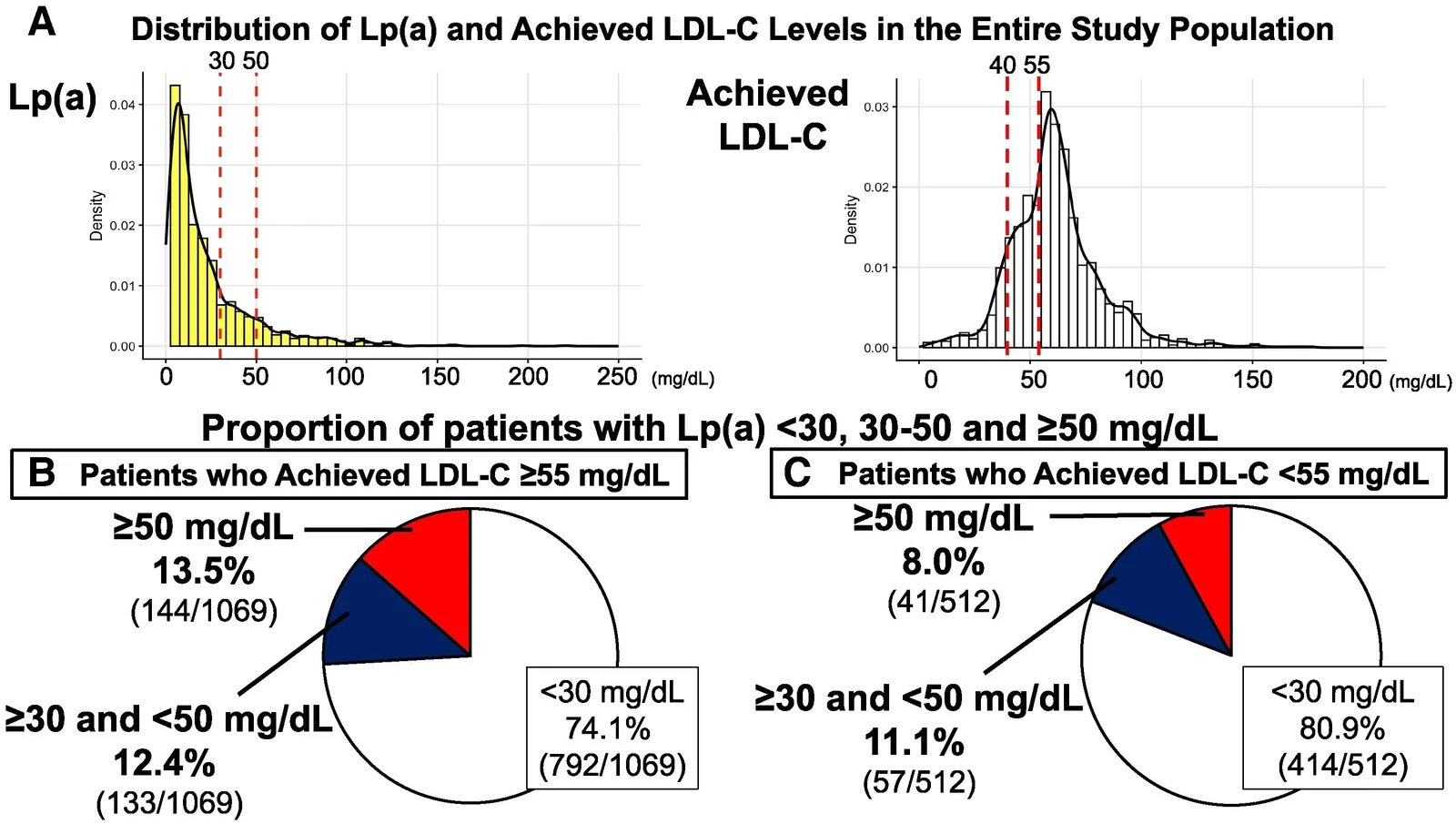
Distributions of Lp(a) levels. (*A*) Distribution of Lp(a) and achieved LDL-C levels in entire study population. (*B*) The proportions of Lp(a) <30, ≥30 and <50, and ≥50 mg/dL in patients with achieved LDL-C ≥55 mg/dL. (*C*) The proportions of Lp(a) <30, ≥30 and <50, and ≥50 mg/dL in patients with achieved LDL-C <55 mg/dL. To convert lipid values to SI units: LDL-C, multiply by .02586 (mg/dL to mmol/L); Lp(a), multiply by 2.381 (mg/dL to mmol/L). LDL-C, low-density lipoprotein cholesterol; Lp(a) = lipoprotein(a)

### Clinical characteristics of patients with achieved LDL-C ≥55 mg/dL

*Table 1* summarizes the comparison of clinical demographics in those stratified according to Lp(a) levels [Lp(a) <30, ≥30 and <50, and ≥50 mg/dL]. Patients with Lp(a) ≥50 mg/dL were older. The frequencies of dyslipidaemia and polyvascular disease were numerically higher in patients with Lp(a) ≥50 mg/dL. Use of statins and high-intensity statins was similar across Lp(a) levels. Patients with Lp(a) ≥50 mg/dL were more likely to be treated with ezetimibe and PCSK9-I, but the latter comparison did not meet statistical significance. As a consequence, LDL-C and triglyceride were lower and HDL-C levels were higher among those with Lp(a) ≥50 mg/dL.

**Table 1:** Clinical demographics in patients with achieved low-density lipoprotein cholesterol ≥55 mg/dL

	Overall (*n* = 1069)	Lp(a) Levels			*P*-value
		<30 mg/dL (*n* = 792)	≥30 and <50 mg/dL (*n* = 132)	≥50 mg/dL (*n* = 145)	
Age (years)	68.8 ± 12.3	68.3 ± 12.4	69.7 ± 12.3	70.9 ± 12.0	.044
Women, *n* (%)	294 (27.5)	204 (25.8)	40 (30.3)	50 (34.5)	.071
BMI (kg/m^2^)	24.1 ± 3.7	24.1 ± 3.7	24.2 ± 3.4	23.9 ± 3.8	.711
Hypertension, *n* (%)	797 (74.6)	589 (74.4)	99 (75.0)	109 (75.2)	.971
Dyslipidaemia, *n* (%)	840 (78.6)	608 (76.8)	111 (84.1)	121 (83.4)	.050
Type 2 diabetes mellitus, *n* (%)	390 (36.5)	287 (36.2)	53 (40.2)	50 (34.5)	.595
Smoking, *n* (%)	112 (10.5)	81 (10.2)	18 (13.6)	13 (9.0)	.404
CKD, *n* (%)^a^	508 (47.5)	368 (46.5)	62 (47.0)	78 (53.8)	.264
Characteristics of CAD, *n* (%)					
ACS	636 (59.5)	468 (59.1)	84 (63.6)	84 (57.9)	.935
CCS	433 (40.5)	324 (40.9)	48 (36.4)	61 (42.1)	
MVD	334 (31.2)	245 (30.9)	42 (31.8)	47 (32.4)	.701
Prior MI	149 (13.9)	104 (13.1)	18 (13.6)	27 (18.6)	.103
Extracoronary artery disease, *n* (%)					
A history of stroke	135 (12.6)	92 (11.6)	21 (15.9)	22 (15.2)	.237
A history of LEAD	57 (5.3)	37 (4.7)	9 (6.8)	11 (7.6)	.256
Polyvascular disease	171 (16.0)	114 (14.4)	28 (21.2)	29 (20.0)	.051
Medication use, *n* (%)					
Statin	1027 (96.1)	757 (95.6)	130 (98.5)	140 (96.6)	.268
High-intensity statin	737 (68.9)	540 (68.2)	93 (70.5)	104 (71.7)	.644
Ezetimibe	627 (58.7)	445 (56.2)	79 (59.8)	103 (71.0)	.003
PCSK9-I	20 (1.9)	13 (1.6)	1 (.8)	6 (4.1)	.075
GLP1-RA	38 (3.6)	29 (3.7)	6 (4.5)	3 (2.1)	.512
SGLT2 inhibitor	79 (7.4)	61 (7.7)	11 (8.3)	7 (4.8)	.432
Metformin	60 (5.6)	43 (5.4)	10 (7.6)	7 (4.8)	.554
Insulin	9 (.8)	7 (.9)	0 (.0)	2 (1.4)	.440
Beta-blocker	612 (57.2)	454 (57.3)	73 (55.3)	85 (58.6)	.853
ACE-I/ARB	675 (63.1)	499 (63.0)	79 (59.8)	97 (66.9)	.472
Biochemistry data					
Lp(a) (mg/dL)	14.7 (7.2–31.0)	9.7 (5.6–17.2)	37.6 (35.0–44.3)	74.1 (58.6–90.8)	<.001
Achieved LDL-C (mg/dL)	65.0 (59.0–77.0)	65.0 (59.0–77.0)	64.0 (60.0–76.0)	69.0 (63.0–81.0)	.001
HDL-C (mg/dL)	48.4 ± 13.2	47.9 ± 13.4	49.6 ± 12.9	50.1 ± 12.3	.033
Triglyceride (mg/dL)	125.0 (88.0–185.0)	130.0 (89.0–189.0)	123.5 (88.5–186.0)	109.0 (80.0–163.0)	.019
HbA1c (%)	6.0 (5.7–6.8)	6.0 (5.7–6.8)	6.1 (5.7–7.0)	6.1 (5.7–6.6)	.827

Data are presented as mean ± standard deviation, median (interquartile range), or *n* (%). *P*-values represent nonparametric tests for trend across Lp(a) categories: Jonckheere–Terpstra test for continuous variables and Cochran–Armitage test for categorical variables. To convert lipid values to SI units: LDL-C, HDL-C, multiply by .02586 (mg/dL to mmol/L); triglycerides, multiply by .01129 (mg/dL to mmol/L); Lp(a), multiply by 2.381 (mg/dL to mmol/L).

ACE-I, angiotensin-converting enzyme inhibitor; ACS, acute coronary syndrome; ARB, angiotensin II receptor blocker; BMI, body mass index; CAD, coronary artery disease; CCS, chronic coronary syndrome; CKD, chronic kidney disease; GLP1-RA = glucagon-like peptide-1 receptor analogue; HbA1c, glycated haemoglobin; HDL-C, high-density lipoprotein cholesterol; LDL-C, low-density lipoprotein cholesterol; LEAD, lower extremity artery disease; Lp)a), lipoprotein(a); MI, myocardial infarction; PCSK9-I, proprotein convertase subtilisin/kexin type 9 inhibitor; SGLT2, sodium–glucose cotransporter 2.

^a^CKD was defined as an estimated glomerular filtration rate <60 mL/min/1.73 m^2^.

### Clinical characteristics of patients with achieved LDL-C <55 mg/dL

In the current study, 32.3% (512 of 1581) of the entire study population achieved an LDL-C <55 mg/dL. *Table 2* describes the comparison of clinical demographics in those with Lp(a) <30, ≥30 and <50, and ≥50 mg/dL. There were no significant differences in the frequency of concomitant traditional risk factors, history of ACS, or presence of multivessel disease among the three groups. With regard to extracoronary artery disease, the prevalence of lower extremity arterial disease and polyvascular disease was significantly higher in those with elevated Lp(a) levels (≥30 and <50 and ≥50 mg/dL). Patients with Lp(a) ≥30 and <50 and ≥50 mg/dL were more likely treated with PCSK9-I. Despite greater use of these lipid-lowering therapies (LLT), achieved LDL-C levels was comparable across the three groups.

**Table 2:** Clinical demographics in patients with achieved low-density lipoprotein cholesterol <55 mg/dL

	Overall (*n* = 512)	Lp(a) Levels			*P*-value
		<30 mg/dL (*n* = 414)	≥30 and <50 mg/dL (*n* = 57)	≥50 mg/dL (*n* = 41)	
Age (years)	68.1 ± 12.4	68.0 ± 12.7	69.9 ± 11.3	66.7 ± 11.0	.422
Women, *n* (%)	80 (15.6)	64 (15.5)	10 (17.5)	6 (14.6)	.906
BMI (kg/m^2^)	24.2 ± 3.6	24.2 ± 3.6	24.2 ± 3.0	24.0 ± 4.1	.939
Hypertension, *n* (%)	373 (72.9)	293 (70.8)	49 (86.0)	31 (75.6)	.049
Dyslipidaemia, *n* (%)	353 (68.9)	280 (67.6)	46 (80.7)	27 (65.9)	.123
Type 2 diabetes mellitus, *n* (%)	209 (40.8)	160 (38.6)	29 (50.9)	20 (48.8)	.118
Smoking, *n* (%)	86 (16.8)	73 (17.6)	10 (17.5)	3 (7.3)	.239
CKD, *n* (%)^a^	242 (47.3)	192 (46.4)	28 (49.1)	22 (53.7)	.643
Characteristics of CAD, *n* (%)					
ACS	316 (61.7)	264 (63.8)	32 (56.1)	20 (48.8)	.111
CCS	196 (38.3)	150 (36.2)	25 (43.9)	21 (51.2)	
MVD	152 (29.7)	124 (30.0)	14 (24.6)	14 (34.1)	.571
Prior MI	63 (12.3)	51 (12.3)	18 (13.6)	27 (18.6)	.213
Extracoronary artery disease, *n* (%)					
A history of stroke	67 (13.1)	49 (11.8)	12 (21.1)	6 (14.6)	.147
A history of LEAD	54 (10.5)	36 (8.7)	12 (21.1)	6 (14.6)	.012
Polyvascular disease	108 (21.1)	75 (18.1)	21 (36.8)	12 (29.3)	.002
Medication use, *n* (%)					
Statin	485 (94.7)	391 (94.4)	54 (94.7)	40 (97.6)	.696
High-intensity statin	386 (75.4)	314 (75.8)	41 (71.9)	31 (75.6)	.813
Ezetimibe	325 (63.5)	263 (63.5)	36 (63.2	26 (63.4)	.998
PCSK9-I	33 (6.4)	21 (5.1)	9 (15.8)	3 (7.3)	.008
GLP1-RA	19 (3.7)	14 (3.4)	4 (7.0)	1 (2.4)	.358
SGLT2 inhibitor	46 (9.0)	37 (8.9)	6 (10.5)	3 (7.3)	.858
Metformin	28 (5.5)	24 (5.8)	2 (3.5)	2 (4.9)	.764
Insulin	4 (.8)	4 (1.0)	0 (.0)	0 (.0)	.621
Beta-blocker	288 (56.2)	227 (54.8)	37 (64.9)	24 (58.5)	.339
ACE-I/ARB	330 (64.5)	259 (62.6)	41 (71.9)	30 (73.2)	.183
Biochemistry data					
Lp(a) (mg/dL)	11.6 (5.8–25.1)	9.1 (4.7–16.1)	39.2 (32.9–42.6)	62.1 (53.2–89.1)	<.001
Achieved LDL-C (mg/dL)	43.0 (37.0–48.0)	43.0 (37.0–48.0)	42.0 (36.0–50.0)	46.0 (42.0–49.0)	.435
LDL-C <40 mg/dL, *n* (%)	165 (32.2)	140 (33.8)	18 (31.6)	7 (17.1)	.091
HDL-C (mg/dL)	48.1 ± 14.0	48.7 ± 14.3	44.6 ± 10.8	46.8 ± 14.1	.033
Triglyceride (mg/dL)	118.0 (82.7–175.0)	120.0 (84.0–179.0)	124.0 (83.0–172.0)	92.0 (68.0–137.0)	.136
HbA1c (%)	6.1 (5.7–6.9)	6.1 (5.7–6.9)	6.4 (6.0–6.8)	6.5 (5.7–7.1)	.009

Data are presented as mean ± standard deviation, median (interquartile range), or *n* (%). *P*-values represent nonparametric tests for trend across Lp(a) categories: Jonckheere–Terpstra test for continuous variables and Cochran–Armitage test for categorical variables. To convert lipid values to SI units: LDL-C, HDL-C, multiply by .02586 (mg/dL to mmol/L); triglycerides, multiply by .01129 (mg/dL to mmol/L); Lp(a), multiply by 2.381 (mg/dL to mmol/L).

ACE-I, angiotensin-converting enzyme inhibitor; ACS, acute coronary syndrome; ARB, angiotensin II receptor blocker; BMI, body mass index; CAD, coronary artery disease; CCS, chronic coronary syndrome; CKD, chronic kidney disease; GLP1-RA = glucagon-like peptide-1 receptor analogue; HbA1c, glycated haemoglobin; HDL-C, high-density lipoprotein cholesterol; LDL-C, low-density lipoprotein cholesterol; LEAD, lower extremity artery disease; Lp(a), lipoprotein(a); MI, myocardial infarction; PCSK9-I, proprotein convertase subtilisin/kexin type 9 inhibitor; SGLT2, sodium-glucose cotransporter 2.

^a^CKD was defined as estimated glomerular filtration rate <60 mL/min/1.73 m^2^.

### Lp(a) levels and MACE in patients with achieved LDL-C ≥55 mg/dL or <55 mg/dL

*Figure 3* and Supplementary Table I describe the occurrence of MACE in patients with achieved LDL-C ≥55 or <55 mg/dL. Of the 1581 patients included in the analysis, 274 (17.3%) experienced a MACE event. A total of 1307 patients (82.7%) were censored: 1283 (81.2%) at the end of the observation period and 24 (1.5%) due to noncardiac death prior to a MACE event. The proportion of competing events was 2.3% (noncardiac death) for the primary outcome of MACE and the secondary outcome of cardiac death and 4.0% (all-cause death) for the secondary outcome of nonfatal MI. In those with achieved LDL-C ≥55 mg/dL, during the observational period (median 5.1 years; interquartile range: 3.1–6.8 years), MACE more frequently occurred with increasing Lp(a) levels (events per 100 person-years: 3.9 events in Lp(a) <30 mg/dL, 7.9 events in Lp(a) ≥30 and <50 mg/dL, 11.0 events in Lp(a) ≥50 mg/dL) (*Figure 3A*). Similarly, in patients with achieved LDL-C <55 mg/dL, during the 4.8-year observational period (interquartile range: 3.3–6.6 years), Lp(a) ≥50 mg/dL was associated with a greater likelihood of experiencing MACE [events per 100 person-years: 1.4 events in Lp(a) <30 mg/dL, 4.7 events in Lp(a) ≥30 and <50 mg/dL, 7.5 events in Lp(a) ≥50 mg/dL] (*Figure 3B*).

**Figure 3:**
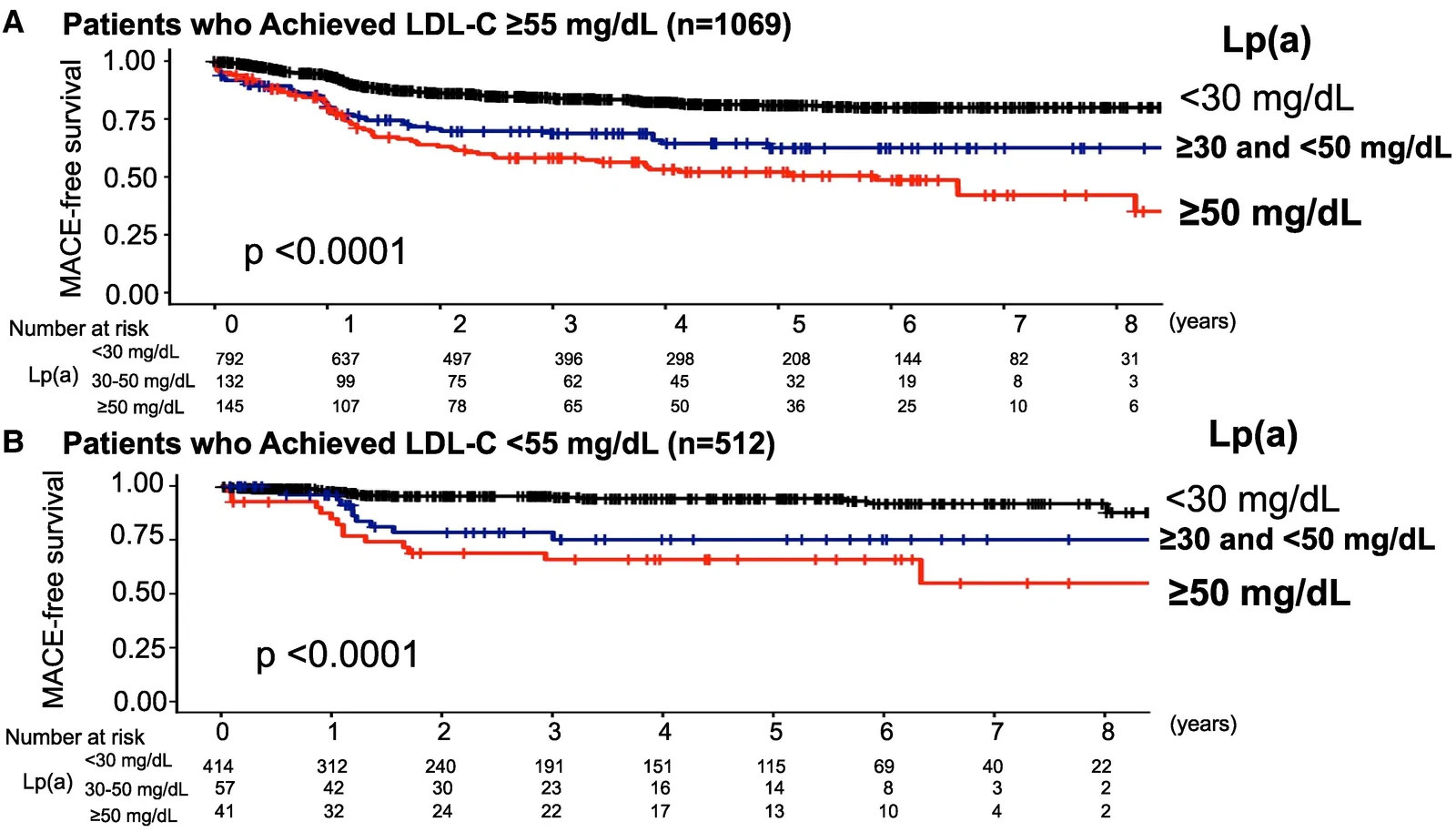
Lp(a) levels and the occurrence of MACE in patients with achieved LDL-C ≥ 55 or <55 mg/dL. (*A*) Patients with achieved LDL-C ≥55 mg/dL. (*B*) Patients with achieved LDL-C <55 mg/dL. To convert lipid values to SI units: LDL-C, multiply by .02586 (mg/dL to mmol/L); Lp(a), multiply by 2.381 (mg/dL to mmol/L). LDL-C, low-density lipoprotein cholesterol; Lp(a), lipoprotein(a); MACE, major adverse cardiovascular event

Supplementary Figures II and III illustrate the comparison of each component of MACE in patients with achieved LDL-C <55 mg/dL or ≥55 mg/dL, respectively. Among both groups, elevated Lp(a) levels were tended to associate with a greater frequency of each component of MACE, although power was lower due to the smaller number of events.

*Figure 4A* shows the comparison of standardized 5-year MACE rates in association with Lp(a) levels (<30, ≥30 and <50, and ≥50 mg/dL) in patients with LDL-C <55 mg/dL or ≥55 mg/dL, respectively. The highest standardized 5-year MACE rates were observed among those with LDL-C ≥55 mg/dL and Lp(a) ≥50 mg/dL (46.9%), while the lowest rate was observed in those with LDL-C <55 mg/dL and Lp(a) <30 mg/dL (5.0%). At every category of Lp(a) levels, standardized 5-year MACE rates were lower in patients who achieved an LDL-C <55 mg/dL compared to those with LDL-C ≥55 mg/dL (*Figure 4A*). However, despite achieving LDL-C <55 mg/dL, standardized 5-year MACE rates still increased in association with increasing Lp(a) levels, rising from 5.0% [Lp(a) <30 mg/dL] to 17.0% [Lp(a) ≥30 and <50 mg/dL] and 33.4% [Lp(a) ≥ 50 mg/dL]. *Figure 4B* shows the fully adjusted HR of MACE with increasing Lp(a) levels (<30, ≥30 and <50, and ≥50 mg/dL) and by LDL-C strata (<55 mg/dL or ≥55 mg/dL), with six mutually exclusive groups and LDL-C <55 mg/dL and Lp(a) <30 mg/dL set as the reference (lowest risk group). Among patients with LDL-C ≥55 mg/dL, the adjusted HRs for MACE were 2.95 (95% CI 1.85–4.71) for Lp(a) <30 mg/dL, 6.95 (95% CI 4.09–11.80) for Lp(a) ≥30 and <50 mg/dL, and 10.34 (95% CI 6.28–17.03) for Lp(a) ≥50 mg/dL, respectively (*Figure 4B*). Even when LDL-C met most guideline recommendations (<55 mg/dL), elevated Lp(a) levels were associated with higher adjusted HRs for MACE compared with the reference group [adjusted HR 3.80 (95% CI 1.78–8.11) for Lp(a) ≥30 and <50 mg/dL and adjusted HR 6.90 (95% CI 3.53–13.46) for Lp(a) ≥50 mg/dL] (*Figure 4B*).

**Figure 4:**
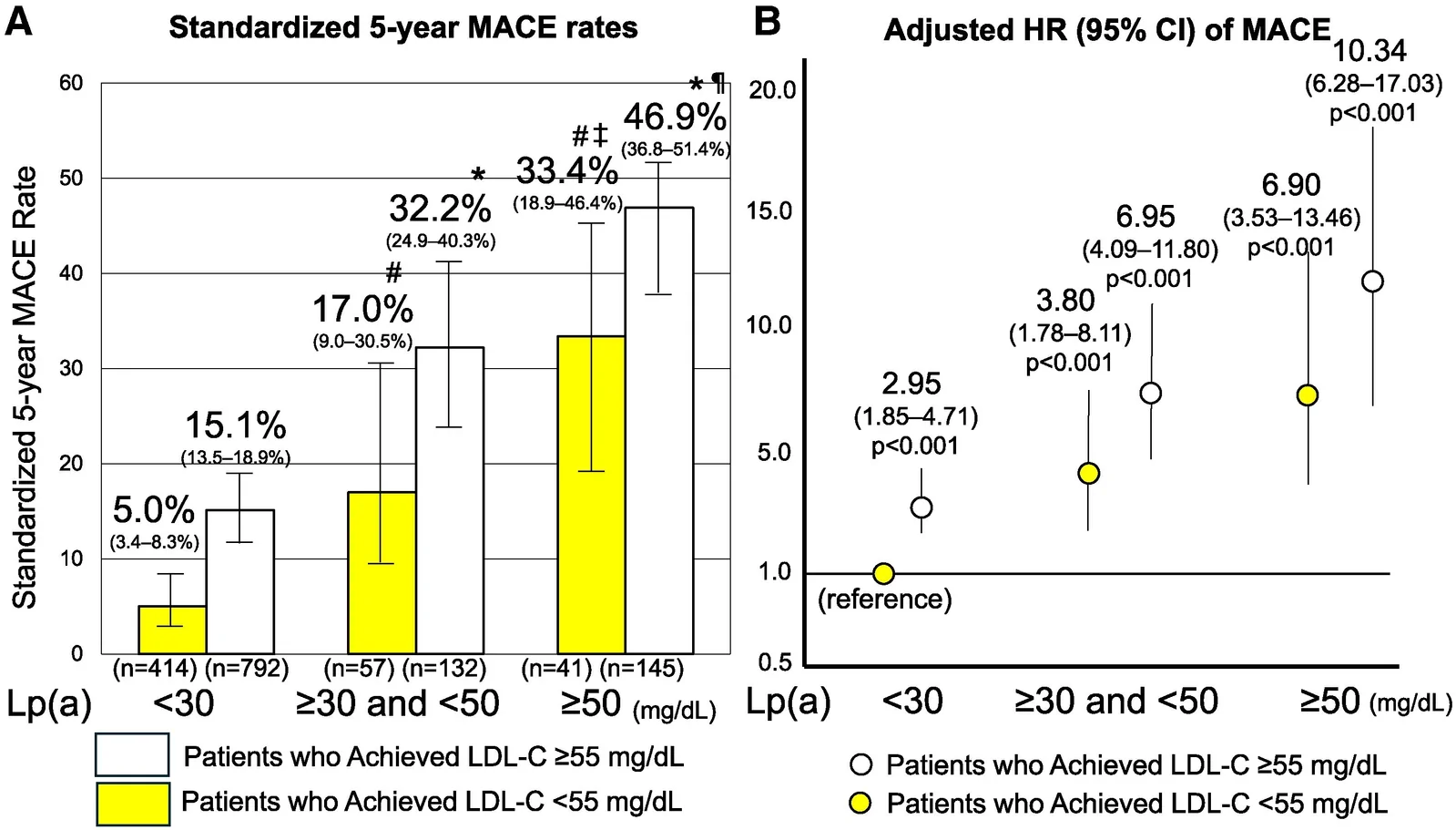
Elevated Lp(a) levels and standardized 5-year MACE rates and adjusted HRs for MACE in patients with achieved LDL-C ≥ 55 or <55 mg/dL. (*A*) Standardized 5-year MACE rates. Standardized 5-year MACE rates across six groups defined by achieved LDL-C levels (<55 and ≥55 mg/dL) and Lp(a) categories (<30, ≥30 and <50, and ≥50 mg/dL), estimated using Cox regression-based marginal standardization (predictive margins). Error bars represent 95% confidence intervals estimated using the bootstrap percentile method (500 resamples). Pairwise comparisons of standardized 5-year MACE rates between groups were performed using bootstrap-based testing (500 resamples). *P*-values were adjusted for multiple comparisons using the Holm method. (*B*) Adjusted HRs (95%CI) for MACE. Adjusted HRs (95%CI) for MACE, estimated from a multivariable Cox proportional hazards model with Group 1 [achieved LDL-C <55 mg/dL and Lp(a) <30 mg/dL] as the reference category. Confidence intervals were based on maximum likelihood estimation. *P*-values represent pairwise comparisons between groups, adjusted for multiple comparisons using the Holm method. Both panels are derived from the same Cox proportional hazards model, adjusted for age, sex, hypertension, Type 2 diabetes mellitus, prior MI, and multivessel disease, with stratification by ACS, dyslipidaemia, and CKD. # *P* < .001 vs. LDL-C <55 mg/dL and Lp(a) <30 mg/dL. ‡ *P* = .008 vs. LDL-C <55 mg/dL and Lp(a) ≥30 and <50 mg/dL. * *P* < .001 vs. LDL-C ≥55 mg/dL and Lp(a) <30 mg/dL. ¶ *P* = .01 vs. LDL-C ≥55 mg/dL and Lp(a) ≥30 and <50 mg/dL. To convert lipid values to SI units: LDL-C, multiply by .02586 (mg/dL to mmol/L); Lp(a), multiply by 2.381 (mg/dL to mmol/L). CI, confidence interval; CKD, chronic kidney disease; HR, hazard ratio; LDL-C, low-density lipoprotein cholesterol; Lp(a), lipoprotein(a); MACE, major adverse cardiovascular event; MI, myocardial infarction

As a sensitivity analysis, we additionally fitted a stratified Cox proportional hazards model with stratification by achieved LDL-C levels (<55 and ≥55 mg/dL), treating Lp(a) as the primary exposure and adjusting for age, sex, hypertension, Type 2 diabetes mellitus, dyslipidaemia, ACS, prior MI, CKD, and multivessel disease. The results were consistent with the primary analysis: compared with Lp(a) <30 mg/dL, the adjusted HRs for MACE were 2.50 (95% CI 1.81–3.45) for Lp(a) ≥30 and <50 mg/dL and 3.91 (95% CI 2.97–5.16) for Lp(a) ≥50 mg/dL, confirming the robustness of our findings.

We formally assessed both multiplicative and additive interactions between Lp(a) and achieved LDL-C levels. The multiplicative interaction was not statistically significant (likelihood ratio test χ^2^ = 1.29, df = 2, *P* = .526). For additive interaction, the RERI was 1.095 (95% CI −.292 to 2.481) for Lp(a) ≥30 and <50mg/dL combined with LDL-C ≥55 mg/dL, which was not statistically significant. However, the RERI was 1.825 (95% CI .539–3.110) for Lp(a) ≥50 mg/dL combined with LDL-C ≥55 mg/dL, indicating a statistically significant positive additive interaction. These findings suggest that the effects of elevated Lp(a) and suboptimally controlled LDL-C on MACE risk are independent and additive rather than multiplicative, indicating that both risk factors contribute separately to cardiovascular risk and that their co-occurrence confers excess risk beyond the sum of their individual contributions.

In the restricted cubic spline sensitivity analysis, Lp(a) was significantly associated with MACE risk in both LDL-C strata (overall *P* < .001 for both). The relationship was approximately linear in patients with achieved LDL-C <55 mg/dL (test for nonlinearity *P* = .057), while a statistically significant nonlinear relationship was observed in patients with achieved LDL-C ≥55 mg/dL (test for nonlinearity *P* < .001) (Supplementary Figure IV). The overall direction and pattern of the association remained consistent with the primary categorical analysis in both strata.

### Lp(a) thresholds and risk of MACE in Japanese patients with CAD

The ROC analyses were conducted to identify thresholds of Lp(a) levels associated with MACE in patients with achieved LDL-C ≥55 mg/dL or <55 mg/dL, respectively (*Figure 5*). In those with achieved LDL-C ≥55 mg/dL, Lp(a) ≥33.8 mg/dL was a cut-off value to predict future risks of MACE [AUC = .63, 95% CI .58–.67, *P* for AUC vs .5 <.001, sensitivity .46 (95% CI .39–.52), specificity .82 (95% CI .79–.84)] (*Figure 5A*). In those with LDL-C <55 mg/dL, the threshold of Lp(a) was 28.2 mg/dL [AUC = .68, 95% CI .58–.77, *P* for AUC vs .5 <.001, sensitivity .56 (95% CI .41–.70), specificity .83 (95% CI .80–.87)] (*Figure 5B*). According to these cut-off values, the proportions of patients with Lp(a) above these thresholds were 23.8% (254 of 1069) and 20.1% (103 of 512) in patients with LDL-C ≥55 mg/dL or <55 mg/dL, respectively.

**Figure 5:**
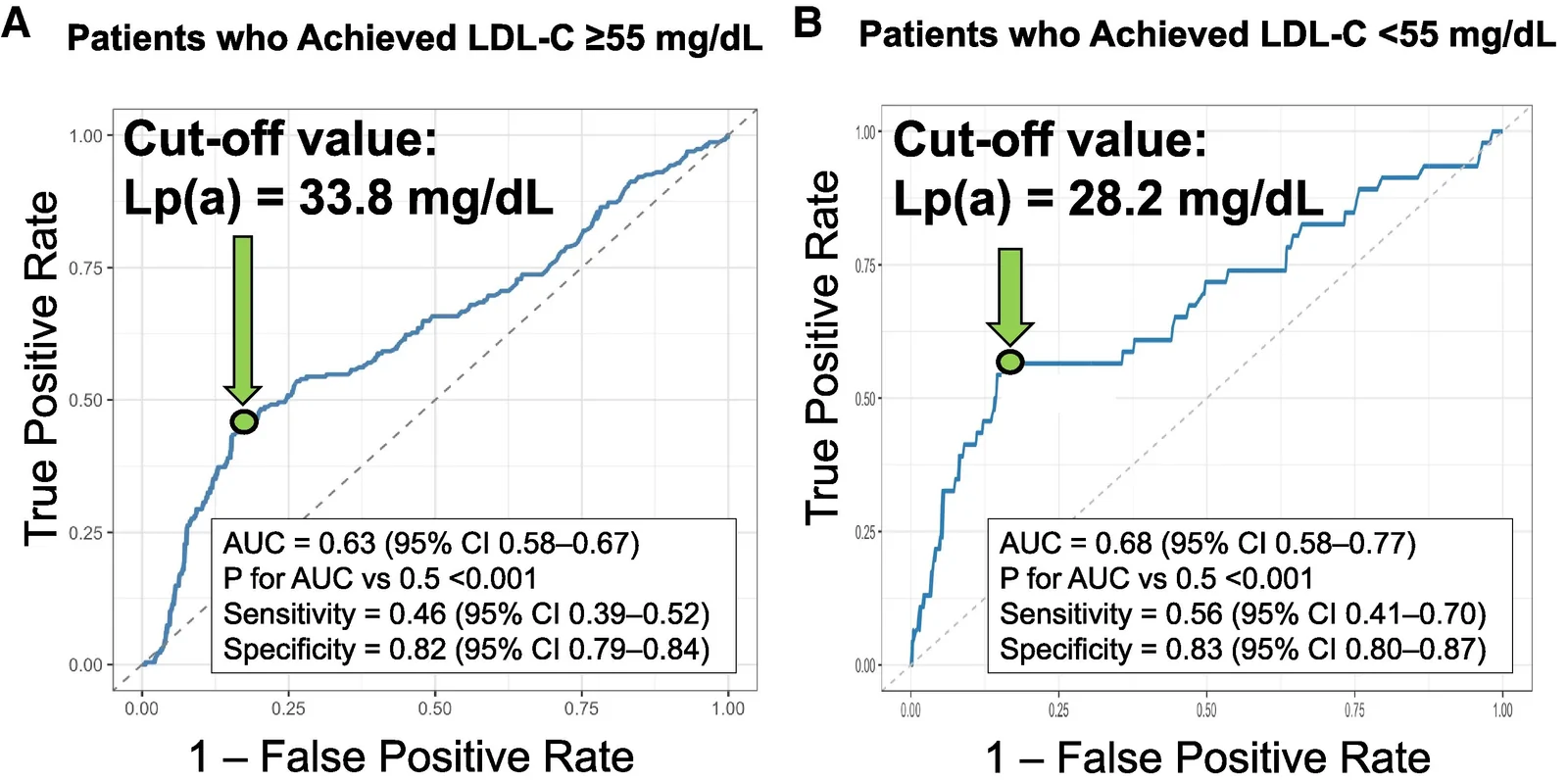
Cut-off values to predict risks of MACE. (*A*) Patients with achieved LDL-C ≥55 mg/dL. (*B*) Patients with achieved LDL-C <55 mg/dL. To convert lipid values to SI units: LDL-C, multiply by .02586 (mg/dL to mmol/L); Lp(a), multiply by 2.381 (mg/dL to mmol/L). AUC, area under the curve; CI, confidence interval; LDL-C, low-density lipoprotein cholesterol; Lp(a), lipoprotein(a); MACE, major adverse cardiovascular event

### Exploratory analyses

#### Lp(a) levels and MACE in patients who achieved LDL-C <40 mg/dL

In the current study, 10.4% (165 of 1581) of patients achieved LDL-C <40 mg/dL after PCI. An exploratory analysis was conducted to investigate the association of Lp(a) levels with risks of MACE in those with achieved LDL-C <40 mg/dL. As shown in Supplementary Figure V, elevated Lp(a) level was still associated with an increased risk of MACE.

#### Association of Lp(a) levels with MACE by sex

In patients with achieved LDL-C ≥55 mg/dL or <55 mg/dL, the association of Lp(a) levels with MACE and its component in men and women is illustrated in Supplementary Figure VI. Across all analyses, there were no significant multiplicative interactions between Lp(a) and sex in patients with achieved LDL-C ≥55 mg/dL or <55 mg/dL, respectively.

#### Lipid-lowering therapies and MACE within each Lp(a) category

The entire study population (*n* = 1581) was stratified into four groups according to LLT (no LLT: *n* = 67; statin alone: *n* = 384; ezetimibe: *n* = 1077; PCSK9-I: *n* = 53). In the statin alone group, 49.9% of them received high-intensity statin. In the ezetimibe group, most of them (99.6%) received the combination of statin and ezetimibe. In the PCSK-I group, all of them was treated with PCSK9-I and statin/ezetimibe. In patients with Lp(a) <30 mg/dL, all three LLT groups were associated with lower MACE risks compared with no LLT: statin alone (HR .16, 95% CI .08–.32), statin plus ezetimibe (HR .21, 95% CI .11–.41), and PCSK9-I (HR .28, 95% CI .09–.82) (Supplementary Figure VII). In contrast, in patients with Lp(a) ≥30 and <50 mg/dL and Lp(a) ≥50 mg/dL, none of the LLT groups demonstrated a significant reduction in MACE risks compared with no LLT.

## Discussion

In the current study which analysed 1581 Japanese patients with CAD, achieving ESC/EAS guideline-recommended LDL-C goals for ASCVD reduced subsequent risk of cardiovascular events, reinforcing the need for adoption of these lower targets in Japan. However, even after attainment of LDL-C goals, elevated Lp(a) levels were still associated with greater frequencies of MACE, suggesting Lp(a) as a key residual risk factor driving future risk. In addition, when Lp(a) levels were high, achieving lower LDL-C levels to <55 mg/dL partially attenuated global cardiovascular risk as currently recommended in the absence of specific Lp(a) lowering therapies. The cut-off values of Lp(a) levels which predicted risk of MACE in patients with achieved LDL-C ≥55 mg/dL or <55 mg/dL was 33.8 and 28.2 mg/dL, respectively (*Structured Graphical Abstract*). These thresholds were lower than that being tested in current ongoing clinical trials of novel Lp(a) lowering agents.

Intensive lowering of LDL-C levels confers greater absolute cardiovascular benefit in patients with high Lp(a) levels. In the sub-analyses of the ODYSSEY Outcomes study, lowering LDL-C levels with alirocumab has reduced rates of first and total cardiovascular events in patients with recent ACS and elevated Lp(a) levels.^29–31^ Furthermore, earlier and greater relative risk reduction of both MACE and limb events was observed in patients exhibiting elevated Lp(a) levels (30 mg/dL or 50 mg/dL).^32^ In our cohort of Japanese patients with CAD, the relationship between achieving lower LDL-C levels and lower risks of MACE was consistently observed in patients with elevated Lp(a) levels. These findings support that lowering LDL-C levels is a still first-line management strategy in patients with elevated Lp(a) levels as recommended by the EAS consensus statement and ESC/EAS guidelines.^10,11^

Lipoprotein(a) is recognized as a residual risk factor even in patients receiving secondary prevention treatment for ASCVD. One recent meta-analysis of statin trials has reported Lp(a) as an independent and continuing risk factor for ASCVD.^33^ In particular, in statin-treated patients who achieved lower LDL-C levels, Lp(a) ≥50 mg/dL identified those who remained at higher risk of MACE.^33^ Pathophysiologically, Lp(a) possesses several proatherogenic properties. As Lp(a) consists of an LDL-like particle carrying cholesterol, it can enter the artery wall, undergo oxidation, and drive foam cell formation as classically observed in atherosclerosis.^1,2^ Additionally, unlike LDL particles, Lp(a) particles carry oxidized phospholipids which may make Lp(a) more atherogenic than LDL particles.^1,2^ These properties of Lp(a) suggest its potential as a driver for promoting atherosclerosis, ultimately leading to clinical manifestations as ASCVD. In the REASSURE-NIRS registry analysing patients who achieved LDL-C <55 mg/dL, lipid-rich plaques still continued to exist in those with Lp(a) ≥50 mg/dL,^34^ which may explain the associations with elevated risk of future cardiovascular events among those with elevated Lp(a).

With the caveat that few patients in our study achieved LDL-C <40 mg/dL and the exploratory nature of such analyses with limited power, we observed that elevated Lp(a) levels were still associated with worse outcomes despite some patients achieving the so-called extreme-risk goals. The sub-analysis from the PACMAN-AMI trial showed that Lp(a) ≥75 nmol/L (≈30 mg/dL) attenuated the efficacy of alirocumab to modulate lipid plaques despite substantially lowering LDL-C levels.^35^ Taken together, these findings further highlight the relevance of Lp(a) on atherosclerosis independent of optimally controlled LDL-C levels and the need to develop specific Lp(a) lowering therapies.

Our sensitivity analyses using restricted cubic splines support the validity of our primary categorical analysis, demonstrating a consistent positive association between Lp(a) and MACE risk across both LDL-C strata (Supplementary Figure IV). In patients with achieved LDL-C <55 mg/dL, the relationship was approximately linear (test for nonlinearity *P* = .057), while in patients with achieved LDL-C ≥55 mg/dL, a significant nonlinear relationship was observed (test for nonlinearity *P* < .001). Although previous large-scale studies have generally reported a linear association between Lp(a) and cardiovascular risk,^3–7^ the apparent nonlinearity in patients with achieved LDL-C ≥55 mg/dL may in part reflect the complex interplay between Lp(a) and residual LDL-C-mediated risk. Furthermore, the significant positive additive interaction between Lp(a) ≥50 mg/dL and LDL-C ≥55 mg/dL (RERI=1.825, 95% CI .539–3.110) supports a synergistic relationship between elevated Lp(a) and suboptimally controlled LDL-C. The apparent attenuation of the association at very high Lp(a) values (>75 mg/dL) should be interpreted with caution given the substantially wider CIs in this range. Taken together, these findings support the validity of our primary categorical analysis.

Ethnicity has a pronounced influence on circulating Lp(a) levels. In the INTERASPIRE and INTERHEART studies, significant differences in Lp(a) concentrations across ethnic groups have been reported.^8,9^ For instance, participants of Chinese (9.8 mg/dL) and Southeast Asian (12.9 mg/dL) ancestry exhibited the lowest level of Lp(a), whereas it was highest in African and Arab participants.^8^ In our Japanese patients with CAD, their median levels of Lp(a) were 12.8 mg/dL, and its threshold to enhance risks of MACE was 33.8 and 28.2 mg/dL in patients with achieved LDL-C ≥55 mg/dL or <55 mg/dL, respectively. Similar to our findings, published studies from Japan consistently reported 15 or 30 mg/dL as cut-off values associated with future cardiovascular events in the secondary prevention settings.^36,37^ Risks of ASCVD were already elevated in those with relatively low Lp(a) levels. As such, cardiovascular risks attributable to Lp(a) perhaps should not be rigidly based on thresholds such as 50 mg/dL. Although the relationship between Lp(a) concentration and cardiovascular disease exists across various ethnicities,^8,9^ they are continuous within each group, and these collective findings underscore the consideration of ethnicity to stratify cardiovascular risk according to Lp(a) levels.

The association of elevated Lp(a) levels with risks of recurrent atherosclerotic cardiovascular events has been reported in patients with a history of ASCVD, regardless of gender and ethnicity.^38^ Additionally, in patients with a history of CAD, higher levels of Lp(a) continued to predict subsequent risks of recurrent ASCVD. These observations and our findings support the importance of Lp(a) levels to predict risks of recurrent ASCVD in the secondary prevention settings.

In patients with achieved LDL-C ≥55 mg/dL or <55 mg/dL, the association of Lp(a) levels with MACE was observed regardless of sex. Published studies have also shown similar risks for MACE associated with higher Lp(a) levels between men and women in both the primary and secondary prevention settings.^38–40^ These collective findings indicate the importance to consider MACE risks attributable to elevated Lp(a) levels in both sexes.

In the current study, any significant reduction in MACE risks with PCSK9-I was not observed in patients with higher Lp(a) levels (Supplementary Figure VII). In contrast to these results, the sub-analysis from the FOURIER study has shown a significant reduction of coronary events with evolocumab in patients with higher Lp(a) levels (≥37 nmol/L [≈88 mg/dL]).^41^ This observation suggests that patients exhibiting elevated levels of Lp(a) may derive greater cardiovascular benefit from potent LLT. Given the very limited number of our study population receiving PCSK9-I, particularly in the Lp(a) ≥30 mg/dL categories [Lp(a) 30–50 mg/dL: *n* = 10; Lp(a) ≥ 50 mg/dL: *n* = 9], further investigations with adequate statistical power are warranted to elucidate the efficacy of potent LLT on cardiovascular outcomes in patients with CAD exhibiting higher Lp(a) levels.

In the current study, guideline-recommended strict LDL-C control was not necessarily sufficient to fully mitigate risk of ASCVD in patients with CAD, indicating the clinical needs for novel agents to specifically modulate Lp(a) levels. Currently, clinical trials are ongoing to test the efficacy of emerging therapies lowering Lp(a) on cardiovascular outcomes. These trials are using single Lp(a) thresholds irrespective of ethnicity, ranging from 150 (≈60 mg/dL) and 175 (≈70 mg/dL) to 200 (≈80 mg/dL) nmol/L.^42–45^ Since Japanese patients with CAD exhibited relatively lower levels of Lp(a) associated with risks of MACE, very limited proportion of them could receive emerging therapies according to Lp(a) criteria in ongoing clinical trials. If ongoing clinical trials show positive results, new trials with lower Lp(a) entry criteria will be needed to investigate the efficacy of agents lowering Lp(a) in different ethnicities, to allow equitable access to therapies and reduce disparities in health outcomes among groups who may be vulnerable to lower Lp(a) levels vs. white Caucasian counterparts.

In Japan, the national PCI registry has reported that around 240 000 Japanese patients with CAD annually receive PCI.^46^ In the current analysis, around 20% of our Japanese patients with CAD presented Lp(a) above threshold associated with risks of MACE. We estimated that almost 20% of Japanese patients with CAD who receive PCI (∼48 000 patients) will have elevated Lp(a) level and hence have a very high subsequent risk of MACE. If the present trials demonstrate the modifiability of Lp(a) and improving outcomes, future trials should be conducted that focus on ethnicities using relatively lower Lp(a) threshold for testing novel agents lowering Lp(a). Such trials would at a global level be better placed to mitigate the residual risk from elevated Lp(a) in the secondary prevention settings, beyond the current suite of trials.

Several caveats should be considered to interpret our findings. First, it was a retrospective study. The selection of lipid-lowering and other medical therapies was not randomized but was decided according to each physician's discretion. The characteristics of our study may have confounded our findings. Second, measurement of Lp(a) levels in units of molar is recommended. However, in the current study, Lp(a) was measured in mass units (mg/dL). Because there is no reliable conversion factor for values obtained with these unit systems, it remains unknown whether Lp(a) levels in molar concentration would result in different findings. Many assays of Lp(a) are hampered by calibration issues, including the isoform dependency of the test procedure and the fact that most individuals are heterozygous for Lp(a) isoforms. Therefore, measurement variability exists attributable to the antibody used in ELISA methods. Third, the approved maximum doses of statis in Japan are lower than those in Western countries. Whether the use of larger doses of statins would affect our findings beyond the recorded achieved LDL-C levels remains uncertain. That said two randomized trials of high-intensity statins vs. lower-intensity statins plus ezetimibe in Korean patients did not show differences in outcomes when similar LDL-C levels were achieved by either approach.^47,48^ Fourth, the findings were derived from patients with CAD who underwent clinically indicated PCI. It is unknown whether these observations can be translated to other types of ASCVD. Fifth, while the current study reported that a lower Lp(a) threshold may be more predictive of MACE in Japanese patients, direct comparison between Japanese and non-Japanese patients with CAD has not been conducted yet. Future investigations are needed to compare the thresholds of Lp(a) for risks of MACE between Japanese and non-Japanese patients with CAD. Sixth, the HRs reported in the present study are subject to the built-in selection bias inherent to Cox proportional hazards models.^49^ As follow-up time progresses, the composition of the risk sets changes due to the differential depletion of susceptible individuals across exposure groups, which may cause the HR to vary over time. The reported HRs should therefore be interpreted as a weighted average of time-varying effects rather than a constant relative risk. Seventh, patients who experienced noncardiac death before a MACE event were censored at the time of noncardiac death, consistent with the cause-specific hazard approach. This approach requires several strong assumptions, including well-defined intervention and conditional exchangeability of censoring, which may not fully hold in observational settings.^50^ Although the proportion of competing events was relatively low in our cohort (noncardiac death: 1.5% of the total cohort), and the impact on our primary findings is therefore expected to be limited, future studies incorporating the subdistribution hazard approach (Fine–Gray model) may provide complementary insights into the absolute risk of MACE in the presence of competing events. Eighth, the subgroup of patients with achieved LDL-C <55 mg/dL had a limited number of events, which may have resulted in imprecise estimates with wide CI.

In conclusion, in Japanese patients with CAD, lowering LDL-C levels was associated with a reduced risk of MACE in those exhibiting elevated Lp(a) levels. This observation supports the current approach to managing patients with high Lp(a) levels. Despite these favourable benefits from LDL-C lowering, elevated Lp(a) remained a substantial residual risk factor for MACE after PCI, indicating the needs for emerging therapies targeting Lp(a). Given that median level and threshold of Lp(a) for MACE risk in Japanese patients with CAD are lower compared to those in Caucasians, a much greater proportion of Japanese patients with CAD may benefit from novel Lp(a) lowering therapies, which are by design excluding a large proportion of the patients studied.

## Supplementary Material

ehag446_Supplementary_Data

## Declarations

### Data Availability

The data underlying this article will be shared on reasonable request to the corresponding author.

### Pre-registered Clinical Trial Number

jRCT1050260016.
