# Supplementary material for: Lipoprotein(a) and residual cardiovascular risks in Japanese patients with coronary artery disease who achieve guideline-recommended low-density lipoprotein cholesterol goals
Source: Eur Heart J. 2026 May 24;47(40):5991–6003. doi: 10.1093/eurheartj/ehag446 (PMC13696080; doi:10.1093/eurheartj/ehag446)
Supplement: Supplementary file 1 [file ehag446_supplementary_data.pdf]

Supplementary Appendix to Lipoprotein(a) and Residual Cardiovascular Risks in Japanese Patients with Coronary Artery Disease who Achieve Guideline Recommended LDL-C Goals

by Yu Kataoka, et al.

Detailed Statistical Analysis……………………………………………………………Page 3-11

Supplementary Figure I. Directed Acyclic Graph (DAG) Representing the Assumed Causal Structure among Variables in the Primary Analysis……………………..…Page 12

Supplementary Figure II. The Occurrence of Each Component of MACE in Patients with Achieved LDL-C ≥ 55 mg/dL…………………………………………………………..Page 13

Supplementary Figure III. The Occurrence of Each Component of MACE in Patients with Achieved LDL-C < 55 mg/dL……………………………………………………..Page 14

Supplementary Figure IV. Continuous Association between Lp(a) and MACE Risk by Achieved LDL-C Levels…………………………………………………………………..Page 15

Supplementary Figure V. Lp(a) Levels and the Occurrence of MACE in Patients with Achieved LDL-C <40 mg/dL…………………………………………………………...Page 16

Supplementary Figure VI. Associations between Lp(a) Levels and MACE Components by Sex……………………………………………………………………………Page 17

Supplementary Figure VII. HRs for MACE by LLT within Each Lp(a) Category…Page 18

Supplementary Table I. Summary of MACE……………………………………..Page 19-20

Detailed Statistical Analysis

Descriptive Statistics

Continuous variables with a reasonably symmetrical distribution are presented as mean (SD), and variables with skewed distributions are presented as median (interquartile range). Symmetry was assessed using the skewness coefficient, with values between -1 and +1 considered indicative of a reasonably symmetrical distribution. Categorical variables are presented as numbers and percentages. Given the ordinal nature of lipoprotein (a) [Lp(a)] categories (<30, ≥30 and <50, and ≥50 mg/dL), all between-group comparisons were performed using non-parametric tests for trend across ordered groups: the Jonckheere-Terpstra test for continuous variables and the Cochran-Armitage test for categorical variables. These non-parametric tests do not require assumptions of normality or homogeneity of variance.

Survival Analysis

Survival was analyzed using the Kaplan-Meier method, and differences between groups were assessed using the log-rank test. The origin for the survival analysis was at 2 months after PCI, which corresponds to the time point at which achieved low-density lipoprotein cholesterol (LDL-C) levels were measured. Patients who experienced a major aderse cardiovascular event (MACE) event within 2 months of percutaneous coronary interventio (PCI) were excluded (Figure 1). Follow-up time started at 2 months after PCI and ended at the date of the first MACE event (cardiac death, non-fatal myocardial infarction, or revascularization), or the last follow-up visit, whichever occurred first. Patients without a MACE event were censored at the last follow-up visit. Patients who experienced non-cardiac death before a MACE event were censored at

the time of non-cardiac death, consistent with the cause-specific hazard approach. Non-cardiac death was defined as all-cause death excluding cardiac death.

Primary Cox Proportional Hazards Model

The association between Lp(a) levels and MACE was assessed using multivariable Cox proportional hazards models. The primary analysis used a six-category exposure variable defined by the combination of Lp(a) levels (<30, ≥30 and <50, and ≥50 mg/dL) and achieved LDL-C strata (<55 and ≥55 mg/dL), with patients having Lp(a) <30 mg/dL and achieved LDL-C <55 mg/dL designated as the reference group (Group 1). Confounders were selected based on the disjunctive cause criterion. Age, sex, chronc kidney disease (CKD), and type 2 diabetes mellitus were identified as common causes of both Lp(a) levels and MACE (confounders). Hypertension, dyslipidemia, acute coronary syndrome (ACS), prior myocardial infarction (MI), and multivessel disease were identified as causes of MACE only and were included to improve the precision of the effect estimate. The assumed causal structure is illustrated in a directed acyclic graph (DAG) provided in Supplementary Figure I.

Assessment of Model Assumptions

The proportional hazards assumption was assessed using Schoenfeld residuals for all covariates included in the model. Age (p=0.119), sex (p=0.795), hypertension (p=0.623), type 2 diabetes mellitus (p=0.445), prior MI (p=0.867), and multivessel disease (p=0.336) satisfied the proportional hazards assumption. However, three covariates (ACS, p=0.008; dyslipidemia, p=0.021; CKD, p=0.010) showed evidence of non-proportional hazards, with a significant global test (p=0.031). These variables were therefore incorporated as stratification factors in the final model using the strata()

function, allowing the baseline hazard to vary across strata. After stratification, the proportional hazards assumption was satisfied for all remaining variables (global p=0.758; primary exposure p=0.380). Importantly, the hazard ratios (HRs) for the primary exposure variable were materially consistent before and after stratification (after stratification vs. before stratification): Group 2: 3.80 [95% confidence interval (CI) 1.78–8.11] vs 3.63 (95% CI 1.70–7.74); Group 3: 6.90 (95% CI 3.53–13.46) vs 6.78 (95% CI 3.48–13.20); Group 4: 2.95 (95% CI 1.85–4.71) vs 2.97 (95% CI 1.87–4.74); Group 5: 6.95 (95% CI 4.09–11.80) vs 6.92 (95% CI 4.08–11.73); Group 6: 10.34 (95% CI 6.28–17.03) vs 10.47 (95% CI 6.38–17.19). All hazard ratios remained statistically significant (all p<0.001), confirming the robustness of our findings. The linearity assumption for the continuous predictor (age) was assessed using restricted cubic splines with 4 knots; the test for non-linearity was not statistically significant (p=0.852), confirming the appropriateness of the linear specification.

Standardized 5-Year MACE Rates

Standardized MACE rates at 5 years were estimated using Cox regression-based marginal standardization (predictive margins) (Figure 4a). The 5-year time point was selected because it approximates the median follow-up duration (median 1,882 days, approximately 5.1 years) while maintaining an adequate number of patients remaining under observation (n=759 at 5 years) and capturing a sufficient number of events (n=236 within 5 years). For each level of the exposure, the individual-level 5-year probability of MACE (π) was predicted for each participant with the exposure set to that level and all other covariates held at their individual observed values (not mean values). These predicted probabilities were then averaged directly on the probability scale

across all individuals to obtain the standardized 5-year MACE rate for each group. The 95% confidence intervals for standardized 5-year MACE rates were estimated using the bootstrap percentile method (500 resamples), in which the 2.5th and 97.5th percentiles of the bootstrap distribution were used as the lower and upper bounds of the 95% CI, respectively. Pairwise comparisons of standardized 5-year MACE rates between groups were performed using bootstrap-based testing (500 resamples). P-values were adjusted for multiple comparisons using the Holm method.

Adjusted Hazard Ratios

Adjusted hazard ratios with 95% CI based on the maximum likelihood estimation for MACE were estimated from the same Cox proportional hazards model, with Group 1 as the reference category (Figure 4b). The primary objective of this analysis was to evaluate the joint prognostic impact of Lp(a) levels and achieved LDL-C on MACE risk; estimating hazard ratios for each combination of Lp(a) and achieved LDL-C strata allows direct quantification of the risk associated with each clinically meaningful combination, which is of direct relevance to therapeutic decision-making. Both the standardized 5-year MACE rates and the adjusted HRs presented in Figure 4 were derived from the same Cox proportional hazards model, adjusted for age, sex, hypertension, type 2 diabetes mellitus, prior MI, and multivessel disease, with stratification by ACS, dyslipidemia, and CKD. Achieved LDL-C was not included as a separate covariate in this model, as it is incorporated into the primary exposure variable (the six-category variable defined by the combination of Lp(a) levels and achieved LDL-C strata). Pairwise comparisons of adjusted hazard ratios between Lp(a) categories

within each achieved LDL-C stratum were performed using separate Cox models for each pair, and P-values were adjusted for multiple comparisons using the Holm method.

Interaction Analyses

We formally assessed both multiplicative and additive interactions between Lp(a) and achieved LDL-C levels. Multiplicative interaction was assessed using the likelihood ratio test comparing models with and without an interaction term. Additive interaction was assessed using the relative excess risk due to interaction (RERI) with 95% confidence intervals estimated by the delta method.

Sensitivity Analyses

As a sensitivity analysis to address the potential limitations of categorizing continuous variables, we additionally fitted a stratified Cox proportional hazards model with stratification by achieved LDL-C levels (<55 and ≥55 mg/dL), treating Lp(a) as the primary exposure and adjusting for age, sex, hypertension, type 2 diabetes mellitus, dyslipidemia, ACS, prior MI, multivessel disease, and CKD.[1] The results were consistent with the primary analysis: compared with Lp(a) <30 mg/dL, the adjusted hazard ratios were 2.505 (95% CI 1.818–3.451) for Lp(a) ≥30 and <50 mg/dL and 3.919 (95% CI 2.972–5.167) for Lp(a) ≥50 mg/dL, confirming the robustness of our findings. The continuous association between Lp(a) and MACE risk was also modeled using restricted cubic splines (RCS) with 4 knots, separately in patients with achieved LDL-C <55 mg/dL and ≥55 mg/dL, using multivariable Cox proportional hazards models adjusted for age, sex, hypertension, type 2 diabetes mellitus, dyslipidemia, ACS, prior MI, multivessel disease, and CKD (Supplementary Figure IV). Overall association and

departure from linearity were assessed using the Wald test. In patients with achieved LDL-C <55 mg/dL, Lp(a) was significantly associated with MACE risk (overall $p<0.001$) with an approximately linear relationship (test for non-linearity $p=0.057$). In patients with achieved LDL-C ≥ 55 mg/dL, Lp(a) was also significantly associated with MACE risk (overall $p<0.001$), although a statistically significant non-linear relationship was observed (test for non-linearity $p<0.001$). The overall direction and pattern of the association remained consistent with the primary categorical analysis, supporting a positive association between Lp(a) and MACE risk across both LDL-C strata.

Exploratory Analyses

As an exploratory analysis, we assessed potential sex differences in the association between Lp(a) as a continuous variable (per 10 mg/dL increase) and each MACE component, separately in patients with achieved LDL-C <55 mg/dL and ≥ 55 mg/dL, using multivariable Cox proportional hazards models adjusted for age, hypertension, type 2 diabetes mellitus, dyslipidemia, ACS, prior MI, multivessel disease, and CKD (sex was not included as a covariate as analyses were conducted separately by sex). Multiplicative interaction between Lp(a) and sex was formally assessed using the likelihood ratio test. No statistically significant multiplicative interaction between Lp(a) and sex was detected in any analysis (all $p>0.05$). In patients with achieved LDL-C <55 mg/dL, results should be interpreted with caution given the limited number of events, particularly for cardiac death (n=4) and MI (n=14). The detailed results are provided in Supplementary Figure VI. To assess whether the association between Lp(a) levels and MACE risk was modified by lipid-lowering therapy, we performed exploratory Cox proportional hazards analyses stratified by Lp(a) category (<30, ≥ 30 and <50, and ≥ 50

mg/dL). Patients were classified into four lipid-lowering therapy groups: (1) no lipid-lowering therapy; (2) statin alone (statin without ezetimibe or PCSK9 inhibitor); (3) statin plus ezetimibe (ezetimibe without PCSK9 inhibitor; 99.6% also received a statin); and (4) PCSK9 inhibitor (regardless of concomitant statin or ezetimibe use). Within each Lp(a) category, HRs for MACE were estimated for each lipid-lowering therapy group relative to the no lipid-lowering therapy group, adjusted for age and sex. The detailed results are provided in Supplementary Figure VII.

Discriminative Ability

The discriminative ability of Lp(a) for predicting MACE was assessed using receiver operating characteristic (ROC) curve analysis. The area under the curve (AUC) with 95% confidence intervals was estimated using the bootstrap method (1,000 resamples). The reported P-value refers to the test of the null hypothesis that the AUC equals 0.5 (no discriminative ability beyond chance), assessed using the Wilcoxon rank-sum test. The optimal cutoff value was determined by Youden's index. Sensitivity and specificity with 95% confidence intervals were estimated using bootstrap resampling (1,000 resamples).

Competing Events

Patients who experienced non-cardiac death before a MACE event were censored at the time of non-cardiac death, consistent with the cause-specific hazard approach. This approach requires several strong assumptions, including well-defined intervention and conditional exchangeability of censoring, which may not fully hold in observational settings.[2] Although the proportion of competing events was relatively low in our cohort (non-cardiac death: 1.5% of the total cohort), and the impact on our primary findings is

therefore expected to be limited, future studies incorporating the subdistribution hazard approach (Fine-Gray model) may provide complementary insights into the absolute risk of MACE in the presence of competing events.

Missing Data

There were no missing values for any of the variables included in the regression models (missing data: 0% for all predictors). All 1,581 participants had complete data (proportion with at least one missing predictor: 0%). Therefore, complete-case analysis was performed, which is equivalent to full-data analysis in this study, and multiple imputation was not required.

Sample Size

As this was a retrospective observational study using an existing registry, a formal a priori sample size calculation was not performed, which is acknowledged as a limitation. The adequacy of the sample size is reflected by the precision of the multivariable-adjusted effect estimates: in patients with achieved LDL-C ≥55 mg/dL, the adjusted hazard ratios for MACE were 2.30 (95% CI 1.61–3.28) or Lp(a) ≥30 and <50 mg/dL vs <30 mg/dL, and 3.51 (95% CI 2.59–4.76) for Lp(a) ≥50 vs <30 mg/dL, indicating adequate precision. In patients with achieved LDL-C <55 mg/dL, the adjusted hazard ratios for MACE were 3.64 (95% CI 1.68–7.92) for Lp(a) ≥30 and <50 mg/dL vs <30 mg/dL and 5.96 (95% CI 3.05–11.67) for Lp(a) ≥50 vs <30 mg/dL; while the confidence intervals were wider reflecting the smaller number of events in this subgroup, the estimates remained statistically significant and directionally consistent with the primary analysis.

A value of $p<0.05$ was considered statistically significant. All statistical analyses were performed using R version 4.5.1 (R Foundation for Statistical Computing, Vienna, Austria).

References

1. Altman DG, Royston P. The cost of dichotomising continuous variables. *BMJ* 2006;332:1080. doi: 10.1136/bmj.332.7549.1080.
2. Mansournia MA, Nazemipour M, Etminan M. A practical guide to handling competing events in etiologic time-to-event studies. *Global Epidemiology* 2022;4:100080. doi: 10.1016/j.gloepi.2022.100080.

Supplementary Figure I. Directed Acyclic Graph (DAG) Representing the Assumed Causal Structure among Variables in the Primary Analysis.

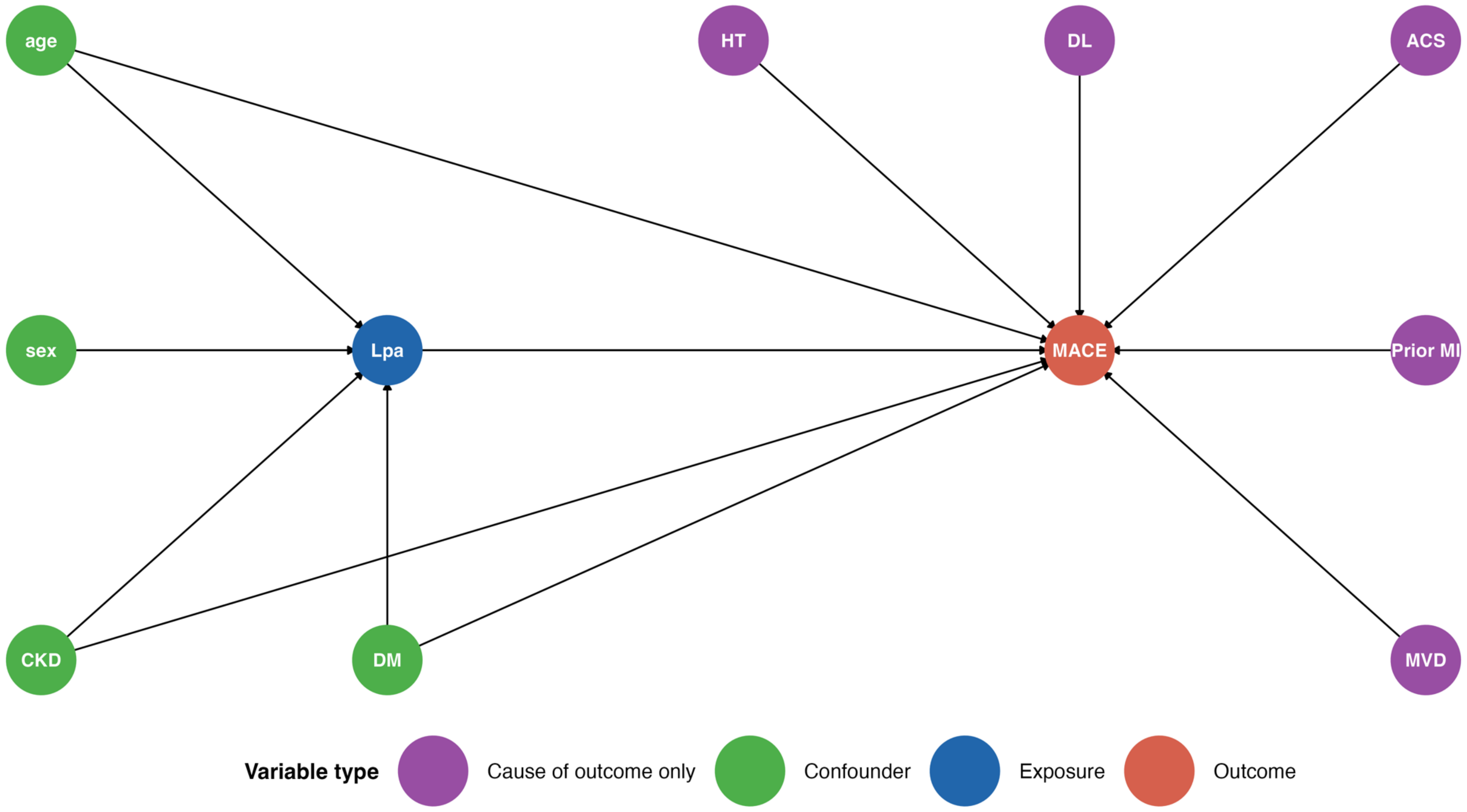

The directed acyclic graph illustrates the assumed causal relationships among variables included in the multivariable Cox proportional hazards model. The primary exposure variable is Lp(a) levels (blue). The outcome is MACE (red). Green nodes represent confounders (common causes of both Lp(a) and MACE): age, sex, CKD, and type 2 diabetes mellitus. Type 2 diabetes mellitus has been shown to reduce Lp(a) levels while independently increasing cardiovascular risk. Purple nodes represent variables identified as causes of MACE only: hypertension, dyslipidemia, ACS, prior MI, multivessel disease. These variables were included in the model in accordance with the disjunctive cause criterion to improve the precision of the effect estimate. Arrows indicate the direction of assumed causal effects.

Supplementary Figure II.
The Occurrence of Each Component of MACE in Patients with Achieved LDL-C ≥ 55 mg/dL

— Lp(a) <30 mg/dL — Lp(a) 30-50 mg/dL — Lp(a) ≥50 mg/dL

(a) cardiac-cause death

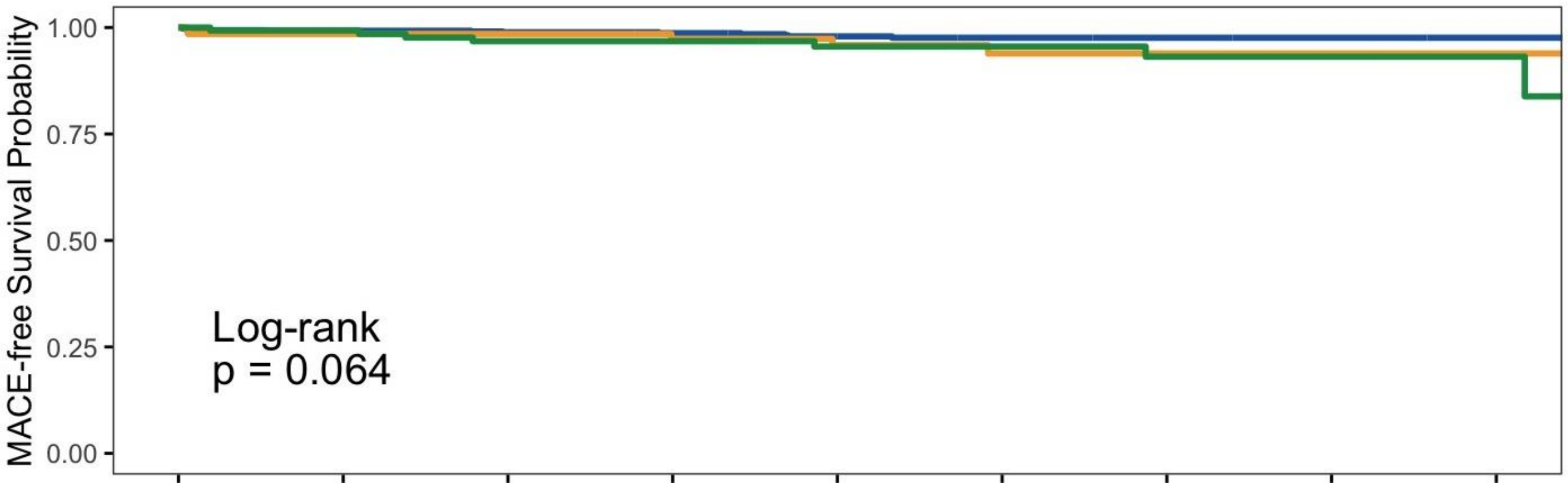

Number at risk Lp(a)	0	1	2	3	4	5	6	7	8 (years)
<30 mg/dL	792	673	565	457	346	243	167	96	37
30-50 mg/dL	132	119	103	84	65	45	29	13	8
≥50 mg/dL	145	127	111	92	75	56	39	20	10

(b) non-fatal MI

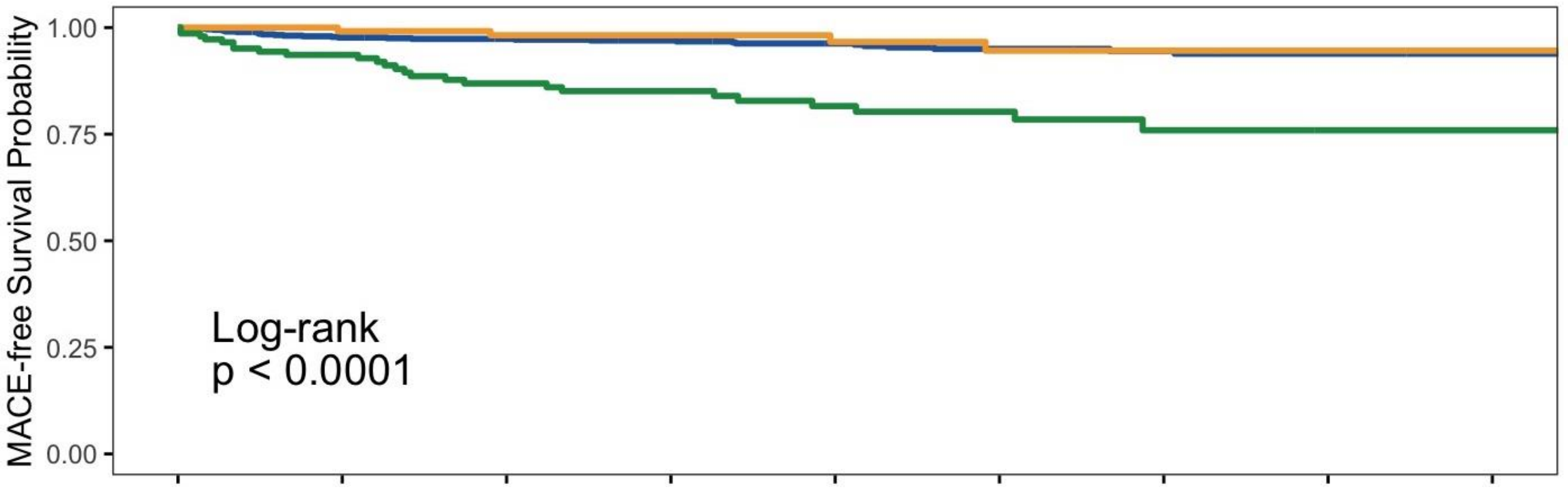

Number at risk Lp(a)	0	1	2	3	4	5	6	7	8 (years)
<30 mg/dL	792	658	551	444	336	232	158	87	32
30-50 mg/dL	132	118	101	82	62	48	27	11	7
≥50 mg/dL	145	121	99	82	65	46	30	14	8

(c) Clinically driven coronary revascularization at non-culprit segment

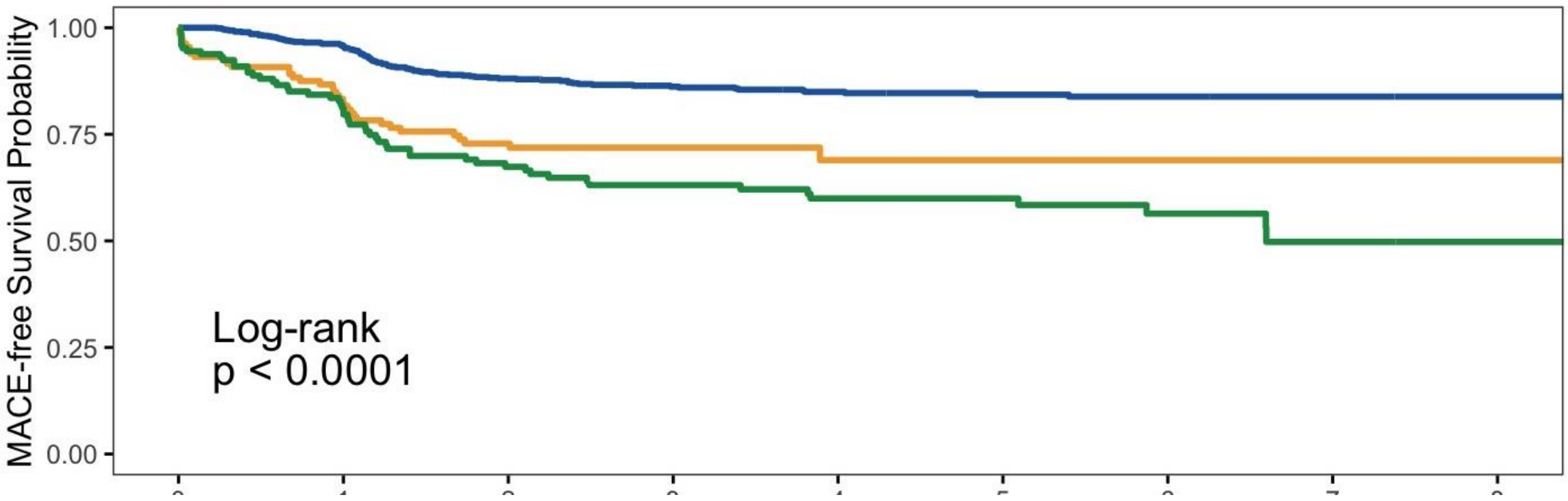

Number at risk Lp(a)	0	1	2	3	4	5	6	7	8 (years)
<30 mg/dL	792	643	501	398	299	211	146	83	30
30-50 mg/dL	132	99	76	63	46	33	20	8	4
≥50 mg/dL	145	105	79	68	54	41	28	13	6

To convert lipid values to SI units: LDL-C, multiply by 0.02586 (mg/dL to mmol/L); Lp(a), multiply by 2.381 (mg/dL to mmol/L).
LDL-C = low-density lipoprotein cholesterol, Lp(a) = lipoprotein (a), MACE = major adverse cardiovascular event

Supplementary Figure III.
The Occurrence of Each Component of MACE in Patients with Achieved LDL-C < 55 mg/dL

— Lp(a) <30 mg/dL — Lp(a) 30-50 mg/dL — Lp(a) ≥50 mg/dL

(a) cardiac-cause death

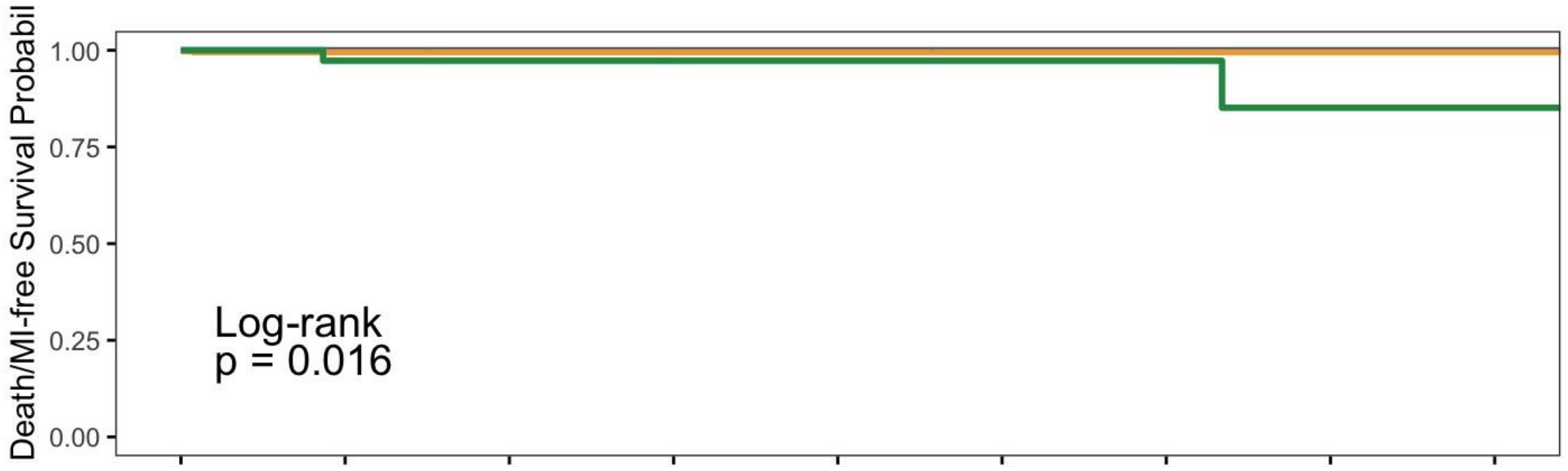

Number at risk Lp(a)	0	1	2	3	4	5	6	7	8 (years)
<30 mg/dL	414	317	248	199	157	119	71	39	22
30-50 mg/dL	57	44	38	28	22	20	12	5	2
≥50 mg/dL	41	36	31	29	24	20	14	5	2

(b) non-fatal MI

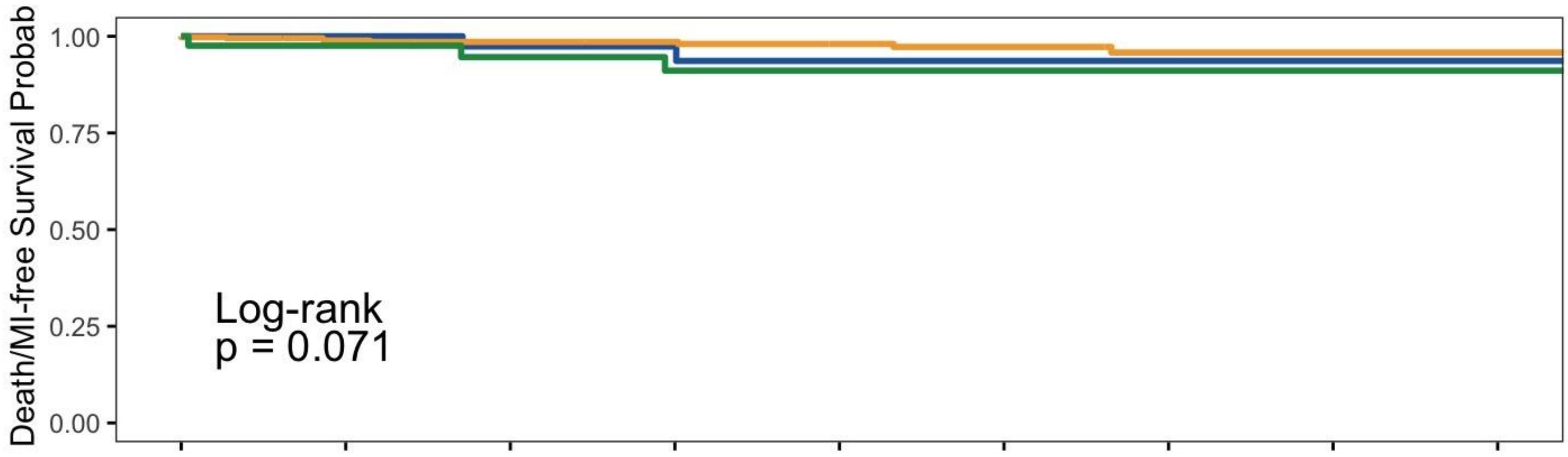

Number at risk Lp(a)	0	1	2	3	4	5	6	7	8 (years)
<30 mg/dL	414	312	235	185	136	95	58	33	21
30-50 mg/dL	57	44	37	26	19	14	7	4	2
≥50 mg/dL	41	35	30	25	21	15	11	4	2

(c) Clinically driven coronary revascularization at non-culprit segment

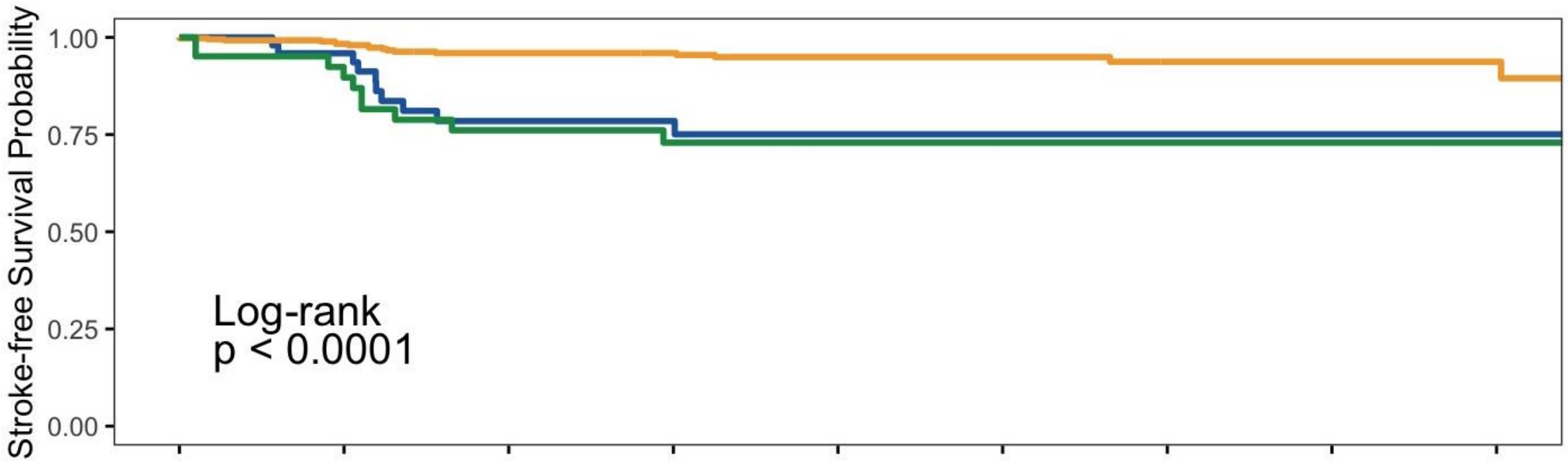

Number at risk Lp(a)	0	1	2	3	4	5	6	7	8 (years)
<30 mg/dL	414	313	240	191	151	115	69	39	22
30-50 mg/dL	57	42	30	23	16	14	8	3	2
≥50 mg/dL	41	33	25	23	18	14	10	4	2

To convert lipid values to SI units: LDL-C, multiply by 0.02586 (mg/dL to mmol/L); Lp(a), multiply by 2.381 (mg/dL to mmol/L).

LDL-C = low-density lipoprotein cholesterol, Lp(a) = lipoprotein (a), MACE = major adverse cardiovascular event

Supplementary Figure IV. Continuous Association between Lp(a) and MACE Risk by Achieved LDL-C Levels

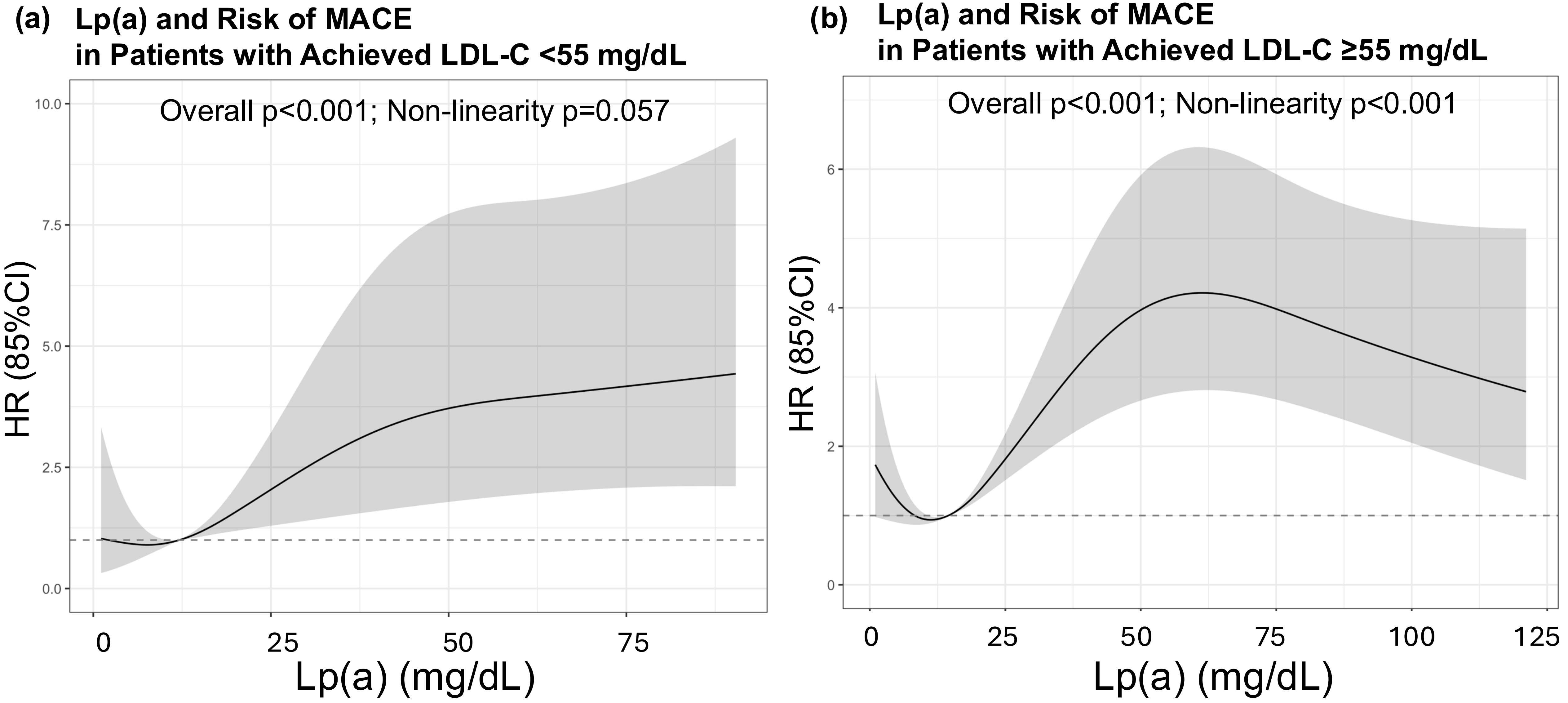

The continuous association between Lp(a) and MACE risk was modeled using restricted cubic splines (RCS) with 4 knots, separately in patients with achieved LDL-C <55 mg/dL(a) and ≥55 mg/dL (b), using multivariable Cox proportional hazards models adjusted for age, sex, hypertension, type 2 diabetes mellitus, dyslipidemia, ACS, prior MI, multivessel disease, and CKD. The reference value is set at the median Lp(a) level. Solid lines represent HR and shaded areas represent 95% CI. Dotted horizontal lines indicate a HR of 1.0. To convert lipid values to SI units: LDL-C, multiply by 0.02586 (mg/dL to mmol/L); Lp(a), multiply by 2.381 (mg/dL to mmol/L).

CI=confidence interval, HR = hazard ratio, LDL-C = low-densiy lipoprotein cholesterol, Lp(a) = lipoprotein (a), MACE = major adverse cardiovascular event

Supplementary Figure V. Lp(a) Levels and the Occurrence of MACE in Patients with Achieved LDL-C <40 mg/dL

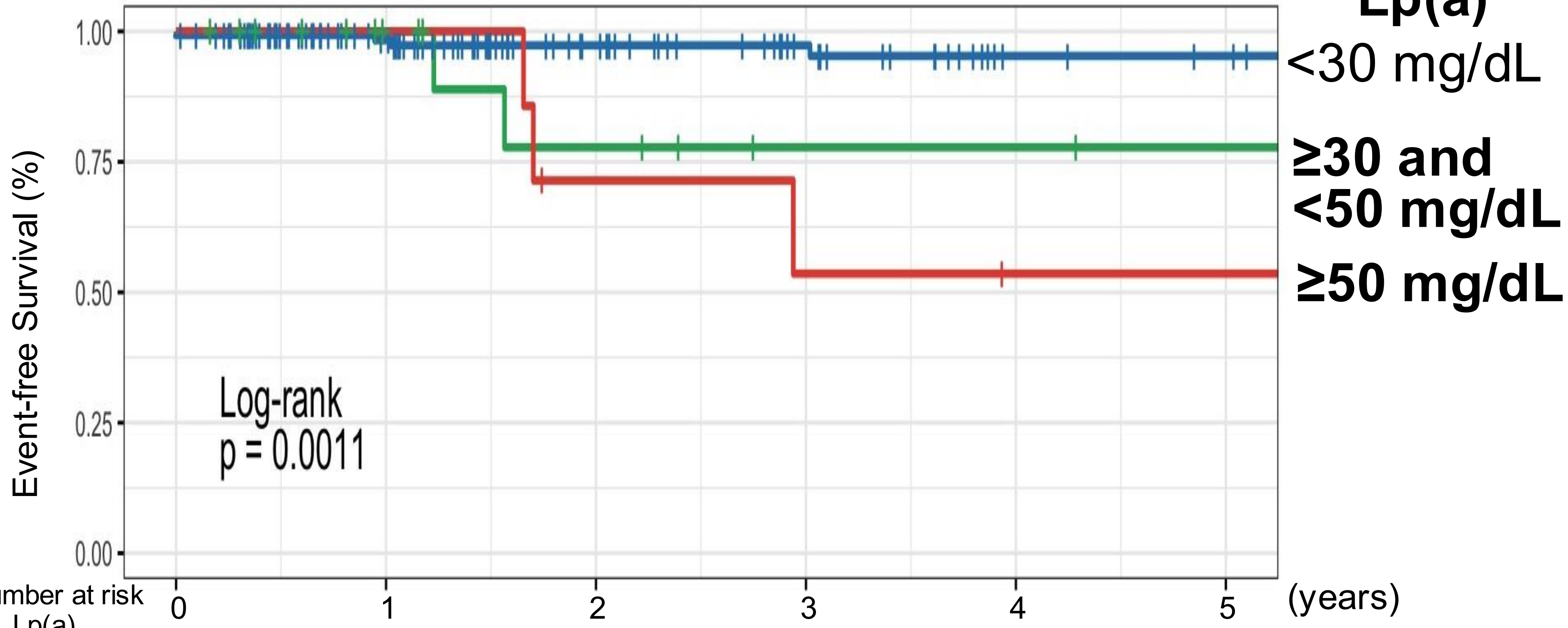

Number at risk Lp(a)	0	1	2	3	4	5
<30 mg/dL	140	100	66	48	33	30
30-50 mg/dL	18	18	17	17	16	16
≥50 mg/dL	7	6	4	2	1	1

To convert lipid values to SI units: LDL-C, multiply by 0.02586 (mg/dL to mmol/L); Lp(a), multiply by 2.381 (mg/dL to mmol/L).
LDL-C = low-density lipoprotein cholesterol, Lp(a) = lipoprotein (a), MACE = major adverse cardiovascular event

Supplementary Figure VI. Associations between Lp(a) Levels and MACE Components by Sex

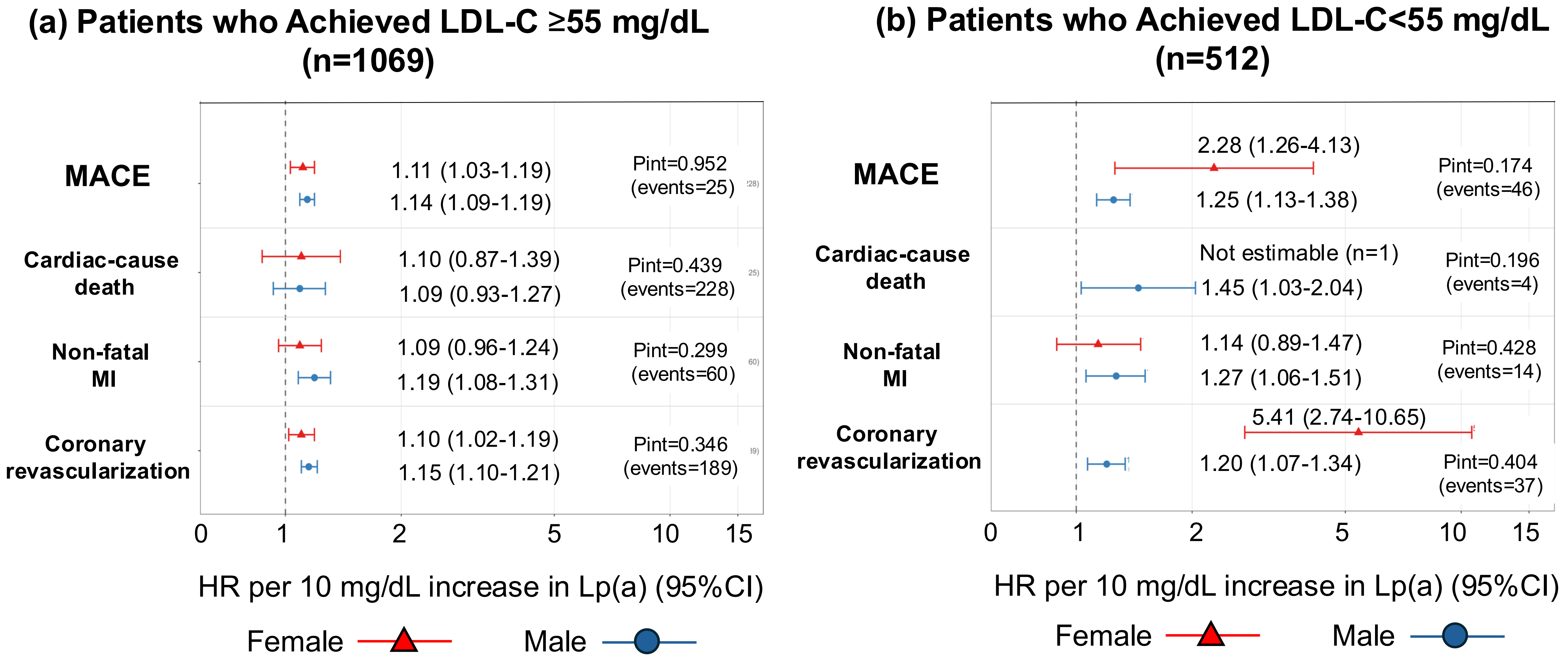

Multivariable Cox proportional hazards models were fitted with Lp(a) as a continuous variable (per 10 mg/dL increase), separately in patients with achieved LDL-C <55 mg/dL and ≥55 mg/dL. Hazard ratios were estimated separately for men and women, adjusted for age, hypertension, type 2 diabetes mellitus, dyslipidemia, ACS, prior MI, multivessel disease, and CKD. Multiplicative interaction between Lp(a) and sex was formally assessed using the likelihood ratio test, comparing models with and without an interaction term between Lp(a) and sex. To convert lipid values to SI units: LDL-C, multiply by 0.02586 (mg/dL to mmol/L); Lp(a), multiply by 2.381 (mg/dL to mmol/L).

CI=confidence interval, LDL-C = low-density lipoprotein cholesterol, HR=hazard ratio, Lp(a) = lipoprotein (a), MACE = major adverse cardiovascular event, MI = myocardial infarction

Pint = P for interaction

Supplementary Figure VII. HRs for MACE by LLT within Each Lp(a) Category

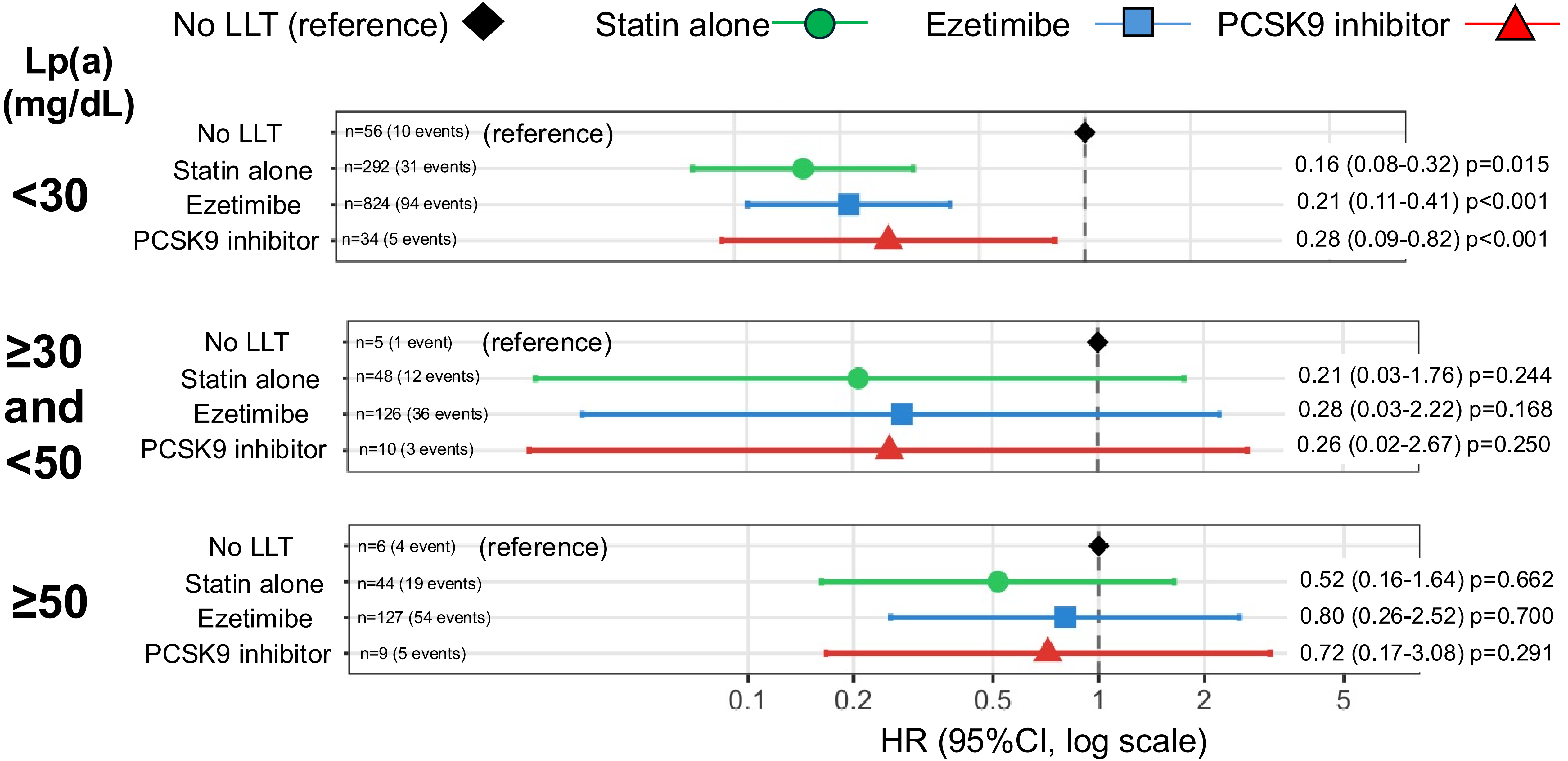

We performed exploratory Cox proportional hazards analyses stratified by Lp(a) category (<30, ≥30 and <50, and ≥50 mg/dL). Within each Lp(a) category, HR for MACE were estimated for each LLT group relative to the no LLT group, adjusted for age and sex. To convert lipid values to SI units: LDL-C, multiply by 0.02586 (mg/dL to mmol/L); Lp(a), multiply by 2.381 (mg/dL to mmol/L).

CI=confidence interval, HR=hazard ratio, LLT = lipid-lowering therapy, Lp(a) = lipoprotein (a), MACE = major adverse cardiovascular event

Supplementary Table I. Summary of MACE

(a) Patients with Achieved LDL-C ≥55 mg/dL

	Overall (n=1069)	Lp(a) Levels		
		<30 mg/dL (n=792)	30-50 mg/dL (n=132)	≥ 50 mg/dL (n=145)
Primary Outcome				
MACE, n (%) (=cardiac death + non-fatal MI + clinically driven coronary. revascularization at non-culprit segments)	228 (21.3)	119 (15.0)	42 (31.8)	67 (46.2)
Secondary Outcomes				
Cardiac death, n (%)	25 (2.3)	13 (1.6)	5 (3.8)	7 (4.8)
Non-fatal MI, n (%)	60 (5.6)	30 (3.8)	4 (3.0)	26 (17.9)
Clinically driven coronary revascularization at non-culprit segments, n (%)	189 (17.7)	97 (12.2)	36 (27.3)	56 (38.6)

(b) Patients with Achieved LDL-C <55 mg/dL

	Overall (n=1069)	Lp(a) Levels		
		<30 mg/dL (n=792)	30-50 mg/dL (n=132)	≥ 50 mg/dL (n=145)
Primary Outcome				
MACE, n (%) (=cardiac death + non-fatal MI + clinically driven coronary. revascularization at non-culprit segments)	46 (4.3)	21 (2.7)	10 (7.6)	15 (10.3)
Secondary Outcomes				
Cardiac death, n (%)	4 (0.4)	2 (0.3)	0 (0.0)	2 (1.3)

Non-fatal MI, n (%)	14 (1.3)	8 (1.0)	2 (1.5)	4 (2.8)
Clinically driven coronary revascularization at non-culprit segments, n (%)	37 (3.5)	17 (2.1)	10 (7.6)	10 (6.9)

To convert lipid values to SI units: LDL-C, multiply by 0.02586 (mg/dL to mmol/L); Lp(a), multiply by 2.381 (mg/dL to mmol/L).

LDL-C = low-density lipoprotein cholesterol, Lp(a) = lipoprotein (a), MACE = major adverse cardiovascular events, MI = myocardial infarction
